# An automated pipeline for extracting histological stain area fraction for voxelwise quantitative MRI-histology comparisons^✰^

**DOI:** 10.1016/j.neuroimage.2022.119726

**Published:** 2022-12-01

**Authors:** Daniel Z.L. Kor, Saad Jbabdi, Istvan N. Huszar, Jeroen Mollink, Benjamin C. Tendler, Sean Foxley, Chaoyue Wang, Connor Scott, Adele Smart, Olaf Ansorge, Menuka Pallebage-Gamarallage, Karla L. Miller, Amy F.D. Howard

**Affiliations:** a Wellcome Centre for Integrative Neuroimaging, FMRIB, Nuffield Department of Clinical Neurosciences, University of Oxford, Headington, Oxford OX3 9DU, , United Kingdom; b Department of Radiology, University of Chicago, Chicago, IL, United States of America; c Academic Unit of Neuropathology, Nuffield Department of Clinical Neurosciences, University of Oxford, Oxford, United Kingdom

**Keywords:** Validation, MRI-histology, Microstructure, Multimodal MRI, Post-mortem

## Abstract

The acquisition of MRI and histology in the same post-mortem tissue sample enables direct correlation between MRI and histologically-derived parameters. However, there still lacks a standardised automated pipeline to process histology data, with most studies relying on manual intervention. Here, we introduce an automated pipeline to extract a quantitative histological measure for staining density (stain area fraction, SAF) from multiple immunohistochemical (IHC) stains. The pipeline is designed to directly address key IHC artefacts related to tissue staining and slide digitisation. Here, the pipeline was applied to post-mortem human brain data from multiple subjects, relating MRI parameters (FA, MD, RD, AD, R2*, R1) to IHC slides stained for myelin, neurofilaments, microglia and activated microglia. Utilising high-quality MRI-histology co-registrations, we then performed whole-slide voxelwise comparisons (simple correlations, partial correlations and multiple regression analyses) between multimodal MRI- and IHC-derived parameters. The pipeline was found to be reproducible, robust to artefacts and generalisable across multiple IHC stains. Our partial correlation results suggest that some simple MRI-SAF correlations should be interpreted with caution, due to the co-localisation of other tissue features (e.g., myelin and neurofilaments). Further, we find activated microglia—a generic biomarker of inflammation—to consistently be the strongest predictor of high DTI FA and low RD, which may suggest sensitivity of diffusion MRI to aspects of neuroinflammation related to microglial activation, even after accounting for other microstructural changes (demyelination, axonal loss and general microglia infiltration). Together, these results show the utility of this approach in carefully curating IHC data and performing multimodal analyses to better understand microstructural relationships with MRI.

## Highlights

• Automated pipeline to generate quantitative maps from immunohistochemical stains.
• Pipeline is generalisable to stains targeting multiple microstructures.
• Pipeline addresses key artefacts related to tissue staining and digitisation.
• Perform voxelwise comparisons, relating microscopy to multimodal MRI.
• Results highlight the importance of analysing multiple stains when validating MRI.

## Graphical abstract

**Figure:**
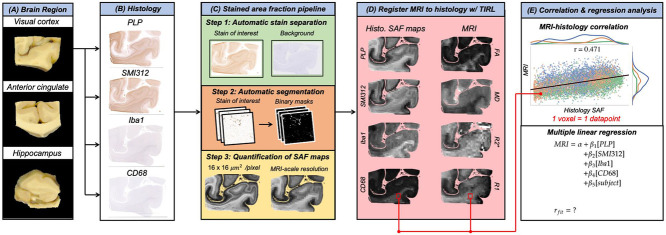

## 1 Introduction

Magnetic resonance imaging (MRI) is a powerful tool that can be used to evaluate neurodegenerative disorders in-vivo. MRI techniques have produced quantitative parameters sensitive to macroscopic neuropathological changes (Johnson et al., 2012; Geraldes et al., 2018; Grolez et al., 2016). However, MRI parameters are non-specific and sensitive to multiple factors related to tissue microstructure. Coupled with millimetre resolution, this leads to difficulty in determining the microstructural underpinnings of a given MRI change (e.g., in disease).

Immunohistochemistry (IHC) is a histological staining technique that can be used to address this difficulty. IHC uses primary antibodies to stain target antigens (proteins related to microstructural features-of-interest) with high specificity. Antigens are then commonly visualised using the chromogen 3′3-diaminobenzidine (DAB) to stain the marked proteins brown. To add contextual information and localise stained tissue features, haematoxylin is often used as a counterstain to mark cell nuclei purple. The acquisition of IHC can aid neuropathological diagnosis (Nave and Werner, 2014; Barker et al., 2013; Ulfig et al., 1998; Atik et al., 2014; Schirmer et al., 2011; Korzhevskii and Kirik, 2016; Jurga et al., 2020; Bagnato et al., 2011; Waller et al., 2019), with antibodies targeting either common tissue features (e.g., myelin or neurofilaments) or markers specific to pathology (e.g., activated microglia, a marker of inflammation (Bachiller et al., 2018; Geloso et al., 2017), or aggregated pTDP-43 in ALS (Pallebage-Gamarallage et al., 2018)). Further, the acquisition of IHC and MRI in the same post-mortem tissue sample enables direct correlation of MR and histologically-derived metrics. This methodology is used to validate MR parameters and improve our microstructural interpretation of MRI. Here, post-mortem MRI functions as a crucial intermediary between IHC and in-vivo imaging. Post-mortem MRI shares a common tissue state with IHC, while possessing the same signal forming mechanisms with in-vivo MRI (Tendler et al., 2022).

While many studies relate IHC to MR parameters (Goubran et al., 2015; Fjær et al., 2013; Fjær et al., 2015; Dusek et al., 2017; Yano et al., 2018; Wang et al., 2020; Abe et al., 2019; Mollink et al., 2019; Lazari and Lipp, 2021), there is still a lack of automated pipelines for extracting quantitative metrics from IHC slides of neuronal tissue (Lazari and Lipp, 2021). Most MRI-IHC analyses rely on heavy manual intervention (van der Weijden et al., 2021; Mancini et al., 2020; De Barros et al., 2019; Seewann et al., 2009), with some using subjective metrics, such as staining intensity scores resembling *low, moderate* or *strong* (Seewann et al., 2009; Bulk et al., 2018). Others directly use the DAB channel's stain density to approximate the amount of targeted protein within the tissue (Seewann et al., 2009; Wiggermann et al., 2017; Hametner et al., 2018; Bagnato et al., 2018). However, this interpretation is limited as the densities do not scale linearly with protein density (De Barros et al., 2019; Hametner et al., 2018; van der Loos, 2008). To circumvent this, other pipelines extract the stain area fraction (SAF) i.e., the number of DAB-stained pixels within a given area. In most pipelines, SAF is quantified by manually setting a threshold for the DAB channel to segment microstructural tissue compartments from non-specific background staining in regions-of-interest (ROIs) (Lazari and Lipp, 2021; van der Weijden et al., 2021; Mancini et al., 2020; De Barros et al., 2019).

The manual derivation of SAF has two main issues. First, the manually-set threshold is dependant on the operator's expertise and a single threshold is often applied to all slides in a dataset or a batch of slides which have been processed together (Pallebage-Gamarallage et al., 2018). Although this is time efficient and avoids intra-observer variability, it comes at the expense of optimising thresholds for individual slides, resulting in decreased robustness to histological artefacts, such as slide-to-slide and within-slide staining intensity variation. Slide-to-slide staining intensity variation is introduced due to unintentional variations in tissue sample preparation and staining, which produce artificial (non-biological) differences in stain intensity and colour information. Within-slide staining artefacts include a gradual staining gradient, with stronger staining at one end of the slide progressing to weaker staining at the other, and striping artefacts from slide digitisation. Staining gradients arise due to how the slide is positioned during staining, inconsistent fixation of the tissue sample, and/or uneven application of the reagent (Suvarna et al., 2019). Striping artefacts describes sharp bands of intensity variation across whole slide images, which arise from when stitching together multiple strips of slide during digitization (Farahani et al., 2015). Though these artefacts could be eliminated at source (e.g., through optimised staining protocols, or an improved slide scanner), they are frequently observed in practice (Suvarna et al., 2019; Farahani et al., 2015; Gurcan et al., 2009; McCann et al., 2015; Madabhushi and Lee, 2016). Further, the impact of these artefacts on the SAF may not always be obvious at the point of slide preparation. Once these impacts are observed, re-staining may be infeasible given the considerable time and manpower already invested. When unaccounted for, these artefacts may impact the extracted IHC metrics. Second, these manual workflows are time intensive. This restricts research studies to smaller sample sizes (i.e., less slides and/or subjects), and limits IHC analyses to hand-drawn ROIs, as opposed to analysing voxels from the whole slide.

Here, we propose an automated SAF pipeline, in the context of an end-to-end MRI-histology workflow, to address these challenges. The automated pipeline is able to extract SAF maps from IHC-stained slides for myelin, neurofilaments and microglia. The pipeline was first evaluated on an IHC dataset designed to test the pipeline's reliability. We then applied the pipeline to a second dataset containing co-registered IHC and MRI to correlate SAF on a voxelwise basis with diffusion-weighted MRI (fractional anisotropy: FA; mean, radial, axial diffusivity: MD, RD, AD) and relaxometry (R2*, R1) maps. To account for covariance between stains, we use partial correlation to identify the unique variance in MRI parameters explained by each targeted protein. Finally, we perform multiple regression with all stains to derive a predictive model of each MR parameter, which may be driven by multiple microstructural sources.

## 2 Data acquisition

We applied the SAF pipeline to two datasets: one with only IHC data, and a second previously published dataset that includes both IHC and co-registered MRI (Pallebage-Gamarallage et al., 2018). Both datasets contain tissue from the same 15 post-mortem brains of patients diagnosed with amyotrophic lateral sclerosis (ALS) and healthy controls (CTL) (12 x ALS, 3 x CTL).

### 2.1 Immunohistochemistry data

After post-mortem MRI, tissue samples were extracted and stained, as described in (Pallebage-Gamarallage et al., 2018), using primary antibodies against PLP (myelin), SMI312 (neurofilaments), Iba1 (microglia) and CD68 (activated microglia, macrophages). These antibodies have applications in pathological and healthy tissue ((Nave and Werner, 2014; Barker et al., 2013) for PLP, (Ulfig et al., 1998; Atik et al., 2014; Schirmer et al., 2011) for SMI312, (Korzhevskii and Kirik, 2016; Jurga et al., 2020; Bagnato et al., 2011; Waller et al., 2019) for CD68 and Iba1) and are relevant to ALS neuropathology, which is characterised by neuronal loss and microglial activation in motor neuron areas (Pallebage-Gamarallage et al., 2018). All antibodies were visualised with DAB and sections were counterstained with hematoxylin. Non-specific staining of endogenous peroxidase—an enzyme present in many cells—was minimised through a peroxidise blocking step (Bussolati and Radulescu, 2011; Del Cerro et al., 1981). Residual non-specific staining was generally faint except for occasional darker stained vasculature that was sparsely distributed across the slides. Slides were digitised with the Aperio ScanScopeⓇ AT Turbo (Leica Biosystems) at x20 object magnification (0.5 µm/pixel). Prior to analysis, each slide was manually quality-checked (QC) where we excluded slides that would fail analysis due to excessive illumination, tears due to poor tissue sectioning or significant amounts of inconsistent staining. Inconsistent staining refers to atypical staining patterns that are non-biological in origin e.g., extremely strong staining gradients, inverted white/grey matter contrasts (opposite to what we neuroanatomically expect) and patchy or unstained tissue.

#### 2.1.1 Evaluation dataset

The “evaluation dataset” was acquired to evaluate our pipeline's performance in terms of reproducibility and robustness to key histological artefacts. Ideally, this data can distinguish true biological variation from variance related to artefacts and analysis. Consequently, data was collected from adjacent tissue slides, which are separated by the slide thickness (6 μm). We assume that adjacent slides have similar microstructure and that the true biological between-slide variance is low. This a priori assumption is reasonable as we aim to summarise microstructure at MRI resolution (0.5–1 mm), which is considerably larger than the slides’ separation. At the tissue boundaries and for proteins-of-interest that sparsely populate the tissue, this assumption may not always be met. For each of the 15 brains, 12–15 adjacent slides were obtained from the primary motor cortex (face region). These slides were separated into groups of 4–5 slides. Each group was stained for CD68, PLP or SMI312. During QC, we removed 49% of the CD68 and 24% of the SMI312 slides. No PLP slides were excluded. CD68 slides were mostly removed due to staining artefacts. This is likely due to some difficulty with this batch's staining, rather than a general issue with the CD68 stain, given that the multiple-region dataset's CD68 slides all passed QC.

#### 2.1.2 Multiple-region dataset

We applied our SAF pipeline (Section 3.1) on the "multiple-region dataset" (Pallebage-Gamarallage et al., 2018) to generate SAF maps. This dataset includes IHC and co-registered MRI data from multiple brain regions with varying levels of disease pathology. Both the dataset and its SAF maps will be available in a future release of the Digital Brain Bank (Tendler et al., 2022). We consider data from the visual cortex (both hemispheres), anterior cingulate (cingulum bundle, corpus callosum) and hippocampus. Slides were stained to visualise CD68, Iba1, PLP and SMI312. All slides passed QC.

### 2.2 MRI data

The data acquisition and pre-processing of the post-mortem MRI have been previously described (Pallebage-Gamarallage et al., 2018; Wang et al., 2020; Tendler et al., 2020). Whole brains were imaged in a 7T human scanner (Siemens Healthcare, Erlangen, Germany) using a 1Tx/32Rx head coil. R2* maps were estimated from susceptibility-weighted data acquired with a 3D multi-echo GRE sequence (parameters: TEs = 2, 8.6, 15.2, 21.8, 28.4, 35 ms with monopolar readout and non-selective RF pulse, TR = 38 ms, flip angle = 15°, bandwidth = 650 Hz/pixel, and in-plane resolution = 0.5 × 0.5 mm^2^ (Wang et al., 2020)). R1 maps were estimated from T1-weighted data collected with a multi-TI turbo spin-echo protocol (Subject 1,2: TE = 14.2 ms, TR = 1000 ms, TIs = 30, 60, 120, 240, 480, 935 ms, flip angles = 180°, bandwidth = 130 Hz/pixel, and in-plane resolution = 1.0 × 1.0 mm^2^ (Pallebage-Gamarallage et al., 2018)). Other brains were imaged with slightly different parameters (Table S1). Diffusion tensor maps of FA, MD, AD and RD (Basser et al., 1994) were estimated from a diffusion-weighted steady-state free precession sequence (parameters: TE = 21.0 ms, TR = 28.0 ms, flip angles = 24°, 94°, bandwidth = 393 Hz/pixel, q value = 300 cm^−1^, number of directions/flip angle = 120, and in-plane resolution = 0.85 × 0.85 mm^2^ (Tendler et al., 2020)).

### 2.3 Co-registration of MRI and histology

MRI and histology data were previously co-registered using FSL's Tensor Image Registration Library (TIRL), which is a general-purpose image registration framework designed for MRI-histology co-registration (Huszar et al., 2019). We used TIRL to 1) register PLP with structural MRI (2D-3D) and 2) co-register other stains to PLP (2D-2D). PLP was chosen as the reference histology data due to its strong white/grey matter (WM/GM) contrast. All MR parameters maps were first aligned to structural MRI using FLIRT (Jenkinson et al., 2002). The generated warps were combined using TIRL to map these MR parameters to the IHC SAF maps for voxelwise correlations. To investigate relationships in WM voxels, WM masks were derived from maps of the third eigenvalue of the diffusion tensor using FSL-FAST (Zhang et al., 2001). WM masks were similarly resampled into the 2D PLP space.

## 3 Methods

We describe an end-to-end workflow for MRI-SAF comparisons. At the centre of this workflow is our automated SAF pipeline, which is described in Section 3.1. The pipeline was designed to be generalisable to multiple IHC stains, reproducible and robust to common IHC artefacts, which we evaluate in Section 3.2. The pipeline was then applied to the multiple-region dataset, which includes IHC and co-registered MRI, facilitating voxelwise MRI-SAF comparisons. We describe how we evaluated the quality of the co-registration (Section 3.3) and performed voxelwise MRI-SAF analyses (Sections 3.4, 3.5) to disentangle the contributions of multiple microstructural features stained with IHC to each MR parameter.

### 3.1 SAF pipeline

The pipeline maps RGB intensity values of high-resolution IHC slides (0.5 µm/pixel) to SAF using three steps: 1) separation of the DAB and haematoxylin stains using colour deconvolution, 2) segmentation of the DAB-stained proteins-of-interest from non-specific DAB using an intensity threshold, and 3) calculation of the SAF map at a specified resolution. The pipeline is automated and data-driven, enabling rapid analysis of many IHC slides across multiple subjects, regions, and stains.

**Fig. 1:**
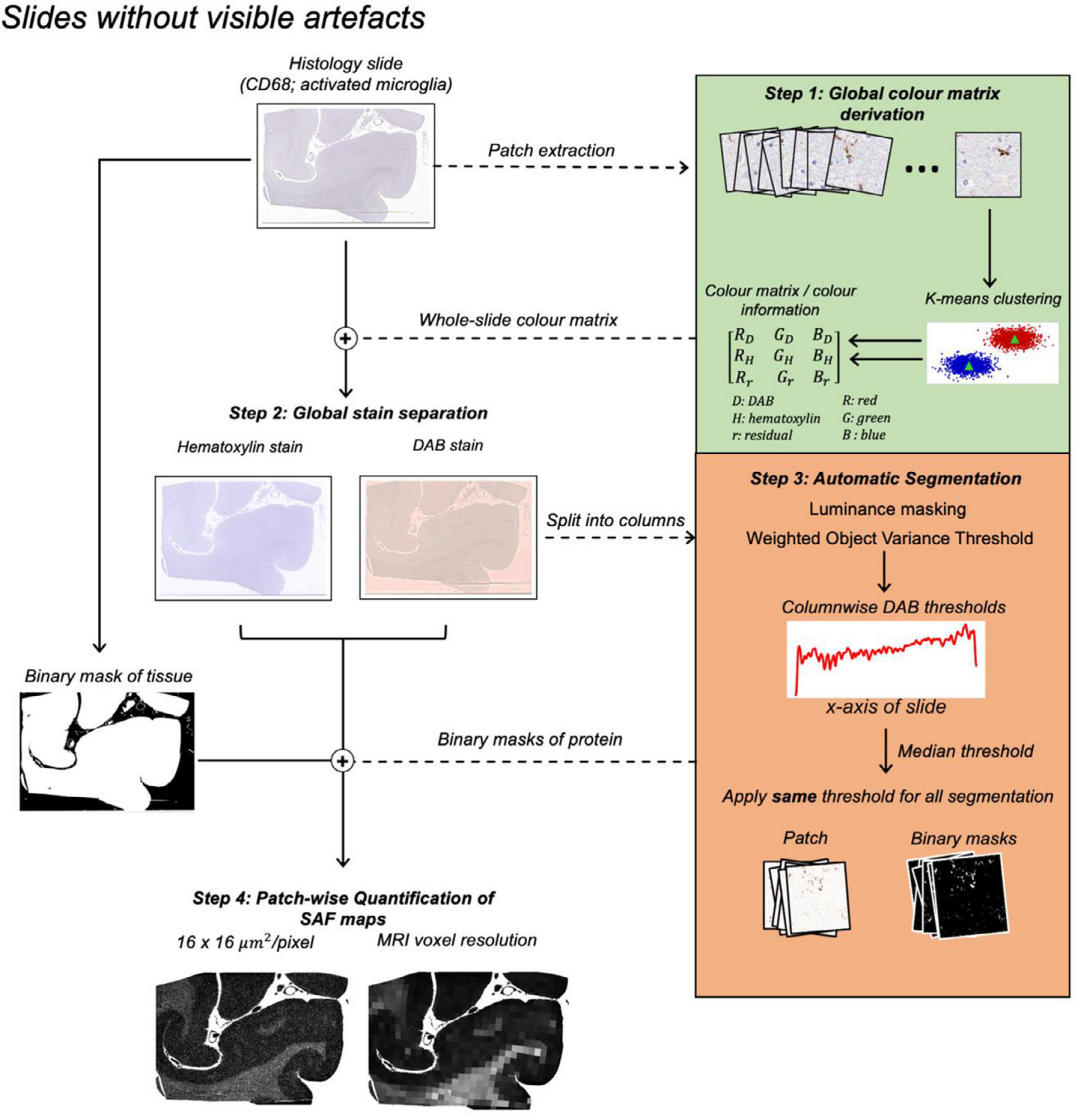
The automated SAF pipeline for stains with little or no intensity gradient and/or visible stitching artefacts (example: visual cortex). This usually includes stains specifying structures that sparsely populate the brain tissue, such as microglia (Iba1, CD68) and neurofilament (SMI312) in some cases. For each slide, the pipeline 1) derives a single, global colour matrix to separate DAB from haematoxylin, 2) performs stain separation to isolate the DAB channel, 3) automatically segments the DAB channel with a single median threshold, and 4) calculates the SAF at variable resolution.

**Fig. 2:**
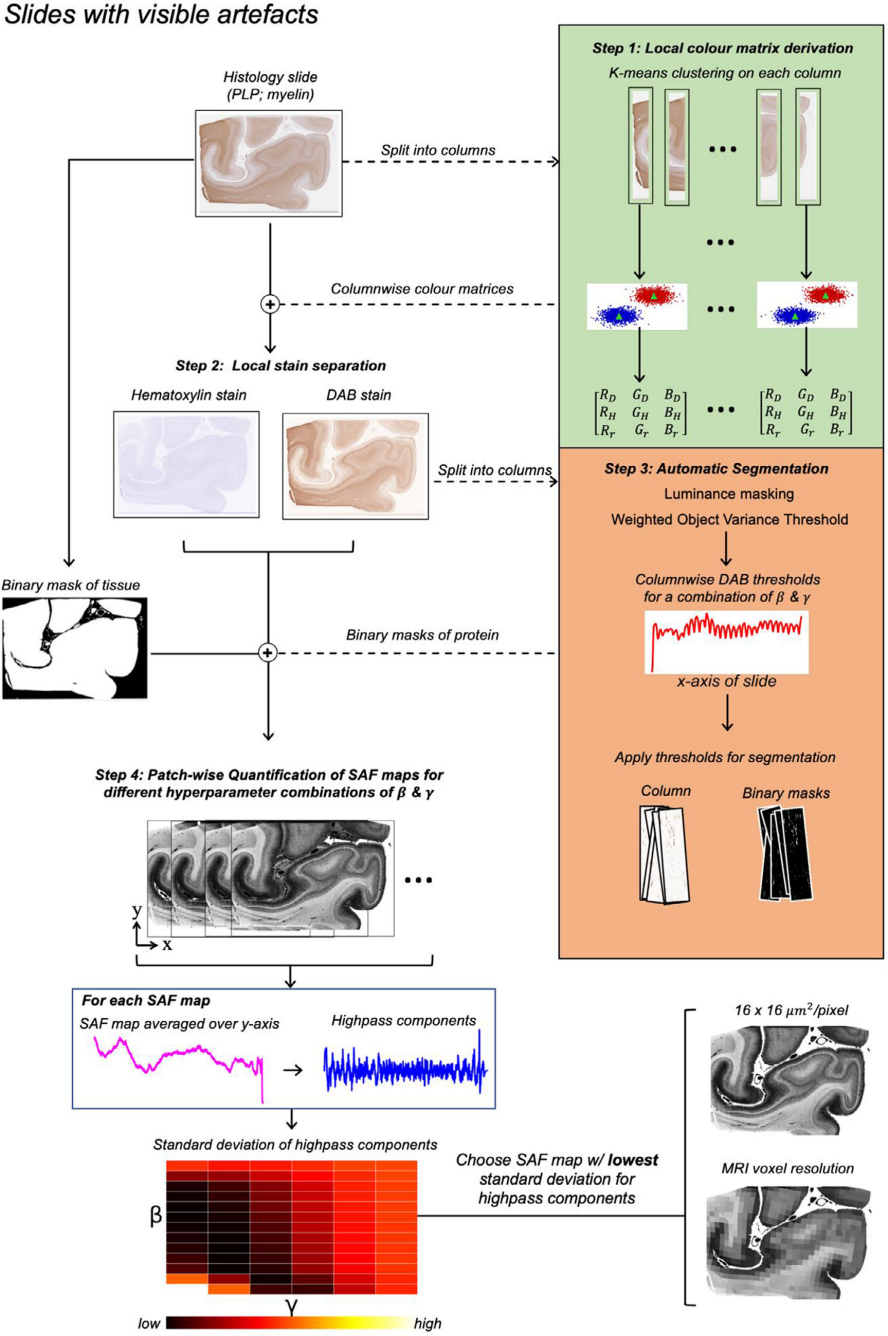
The automated SAF pipeline for stains confounded by staining gradient and/or visible striping artefacts (visual cortex). For each slide, the pipeline 1) derives multiple, columnar colour matrices from the data, 2) performs stain separation for each column, 3) automatically segments each column's DAB channel and 4) forms an SAF map. Steps 3 and 4 are repeated for a range of hyperparameters **β** and **γ**, which are optimised via grid-search to account for within-slide artefacts (Section 3.1.2). **β** modulates how much correction is needed to offset the impact of the striping artefact, while **γ** is a smoothing kernel required to prevent abrupt column-to-column correction. For each SAF map, we averaged the map over its height (y-axis) and chose the SAF map with the lowest standard deviation (i.e., least impacted by these artefacts).

In practice, we found considerable impact of IHC artefacts in some slides. This motivated the development of two “configurations” of the pipeline: the default (Fig. 1) and artefact (Fig. 2) configuration. The default configuration is designed to emulate an expert histologist when deriving SAF (but with data-driven, slide-specific stain separation and thresholding) for IHC slides with no prominent staining gradient and/or striping artefacts. This improves upon a more manual approach, where the adjustment of slide-specific thresholds would be extremely time-consuming. If these artefacts are present, we propose the artefact configuration, which automatically adjusts the local thresholds within-slide to account for the impact of these artefacts. Both configurations are automated, and differ based on whether local or whole-slide methods are used for Steps (1) and (2). We now describe each step of the pipeline in detail.

#### 3.1.1 Colour matrix derivation for stain separation

Colourimetric analysis is based on colour deconvolution, a stain separation method using Beer-Lambert's law (Ruifrok and Johnston, 2001). In Beer-Lambert's law (c.f. Appendix A), the light absorbence (**A**) for RGB channels is linearly related to the stain density (**C**) via the attenuation coefficients (**ε**). The stain (i.e., DAB, hematoxylin) density in a slide can be computed with a matrix inversion:

(1) $$\begin{array}{ccc} \left[\begin{array}{c} C_{DAB}\\ C_{hematoxylin}\\ C_{residual} \end{array}\right] & = & {\left[\begin{array}{ccc} \epsilon_{DAB,red} & \epsilon_{DAB,green} & \epsilon_{DAB,blue}\\ \epsilon_{hema,red} & \epsilon_{hema,green} & \epsilon_{hema,blue}\\ \epsilon_{res,red} & \varepsilon_{res,green} & \epsilon_{res,blue} \end{array}\right]}^{-1}\left[\begin{array}{c} A_{red}\\ A_{green}\\ A_{blue} \end{array}\right]\\ & = & M^{-1}\left[\begin{array}{c} A_{red}\\ A_{green}\\ A_{blue} \end{array}\right] \end{array}$$

where M, termed the colour matrix, is a 3 × 3 matrix with each element giving the attenuation coefficient ($\epsilon_{i,\lambda }$) for each stain (i) and colour (λ) pair. **M** can be defined from literature or empirically measured from single-stained slides (Ruifrok and Johnston, 2001; Landini et al., 2021). Using an **M** that is non-specific to each slide leads to poor stain separation and consequently, inconsistent interpretation during slide-to-slide comparison (Gurcan et al., 2009; Madabhushi and Lee, 2016; Clarke and Treanor, 2017). We address this problem by employing a *k*-means clustering approach to derive colour information directly from the IHC data. This approach is tailored according to the different configurations.

**Default configuration**

To derive a single slide-specific **M**, we used the *k*-means clustering strategy introduced in (Geijs et al., 2018). We randomly sampled patches (0.5 × 0.5 mm^2^; *n* = 200) from the slide and performed *k*-means clustering (*k* = 2) to produce 2 cluster centroids corresponding to the colour vectors (i.e., rows in **M**) of hematoxylin and DAB. We then performed a second *k*-means clustering (*k* = 2) on the 200 colour vectors output from the first *k*-means. This produced two centroids corresponding to the slide-specific DAB and haematoxylin colour vectors of **M**. Stain separation was performed using this single **M** across the whole slide.

**Artefact configuration**

In slides with a staining gradient, a locally changing **M** is required to account for colour differences from one end of the slide to the other. Consequently, we apply *k*-means locally to derive **M** along the gradient direction. In principle, **M** can be derived in windows of pixels of any arbitrary shape (i.e., square patch) that are large enough to provide a sufficient number of pixels of each class (background versus stain-of-interest) for data-driven analysis. In our datasets, the staining gradient was present along the horizontal axis of the slide. Hence, we applied *k*-means on a column-wise basis (32-pixels width; height matching the slide) to define **M_column_** that varies along the gradient direction. Stain separation was performed locally using **M_column_**.

For stain separation in both configurations, an approach emulating NNLS (non-negative least squares) (c.f. Appendix A), was used to estimate the DAB channel's stain density (**C_DAB_**) according to Eq. (1). **C_DAB_** was then converted to intensity values (**I**) using an equation analogous to the inverse Beer-Lambert's Law: ***I*** = 10^−^**^C^_DAB_**.

#### 3.1.2 Automatic thresholding for protein segmentation

To segment the DAB-stained protein-of-interest from non-specific DAB, manual analyses require an expert to manually set a threshold. We improve on this by deriving a data-driven threshold. An image segmentation approach is Otsu's method (Otsu, 1979), which computes a threshold that maximises the image histogram's inter-class variance. Our algorithm is based on the related weighted object variance algorithm (Yuan et al., 2015), which includes a tunable parameter (**δ**) to separate classes of unequal count and variance. Here, we computed a threshold **t** by maximizing:

(2) $$t=\arg\max_{t}\left[P_{0}\left(t\right)\mu_{0}{\left(t\right)}^{2}+P_{1}{\left(t\right)}^{1+\delta }\mu_{1}{\left(t\right)}^{2}\right]$$

Here, **P_j_(t)** and **μ_j_(t)** are the cumulative probabilities and means of the uneven classes ***j*** = 0 (protein-of-interest) and ***j*** = 1 (DAB-stained background). **δ** ranges from −1 to 1 and weights the object variance to shift **t** closer to the mean of the smaller (**δ** > 0) or larger (**δ** < 0) class 0. **δ** < 0 was used for densely stained PLP or SMI312 and **δ** > 0 for sparsely stained slides (CD68, Iba1). To achieve local thresholding, the algorithm was performed on contiguous columns rather than the entire image. Our slides possessed left-to-right gradients in stain density, with vertical bands of intensity (striping). Thresholds **t_i_** were thus calculated on a column-wise basis (32-pixels width; height matching the slide), with **i** representing the column index. Before calculating the threshold, the DAB channel was masked to remove the non-tissue pixels in the column (luminance < 0.75) to balance the two classes in the DAB channel histogram. This prevented the large number of non-tissue pixels from skewing the histogram and biasing the threshold.

**Default configuration**

A single threshold was sufficient to segment the entire slide without visible striping artefacts. The fixed threshold was calculated as the median of all local thresholds computed column-wise. Consequently, our data-driven pipeline replaces expert determination of the threshold, **t**, with determination of the hyperparameter, **δ**. Ideally, this hyperparameter is set once by an expert for a stain. Our pipeline will then enable automated, adaptive thresholding on new slides. In this work, we sampled 8–10 patches (0.5 × 0.5 mm^2^) spanning different brain regions, tissue types and subjects to choose a **δ** (per stain) that produced optimal segmentation. Segmentations were vetted with an expert histologist (MPG).

**Artefact configuration**

In slides with striping artefacts, a single whole-slide threshold is insufficient for optimal segmentation. Our artefact configuration uses column-wise thresholds that adapt to these artefacts in a data-driven way. Columns affected by striping are characterised by DAB histograms with decreased median absolute deviation (MAD) compared to columns less affected by striping artefacts. This was found by empirically comparing histograms from striping and non-striping regions and associating it with an observed slight blurring of the image along the stitching boundary of the slide scanner. Consequently, we use the MAD from each column (indexed with **i**) to weight the **δ** to account for how much striping artefact is present:

(3) $$\delta =\alpha {\left(1+\Delta_{i}\right)}^{\beta }$$

where:

(4) $$\Delta_{i}=\frac{MAD_{i}-\sigma \left(MAD_{i}\right)}{\sigma \left(MAD_{i}\right)}$$

**δ_i_** is the overall exponent used in WOV equation (Equation 2); **α** is a stain-specific value chosen manually which depends on the relative positive stain and background staining (equivalent to **δ** in the default pipeline); **β** is chosen via grid search and depends on amount of striping artefact i.e., it modulates how much **δ** differs from **α** due to striping; **σ** is the kernel size of a 1D Gaussian filter applied to **MAD_i_** (**σ**=16); **Δ_i_** represents the **i^t^**^**h**^ column's change in structure (MAD) relative to neighbouring columns. After calculating the column-wise thresholds using Eqs. 2-4, we performed a final smoothing operation:

(5) $$t_{final}=\gamma \left(t_{i}\right)$$

where **γ** is a 1D Gaussian filter that it optimised via grid search that prevents the thresholds (**t_i_**) from varying abruptly column-to-column.

Figures S1 and S2 demonstrate how the hyperparameters **α, β** and **γ** affect the resultant SAF map. **α** is chosen manually, **β** and **γ** are optimised for each slide via grid search to minimise the standard deviation of highpass components (defined below) in the resulting SAF. In our multiple-region dataset, We observed stitching artefacts and/or staining gradients in the PLP slides only. Hence, we applied the artefact configuration on our PLP slides (**α**=−0.60) and the default configuration on our CD68 (**δ**=0.05), Iba1 (**δ**=0.05) and SMI312 (**δ**=−0.30) slides. Figure S3 shows example segmentations.

#### 3.1.3 Calculating the SAF map

Following segmentation, we computed SAF maps at various resolutions. First, we extracted a tissue mask by applying Otsu's method (Otsu, 1979) on the hematoxylin channel followed by several morphological operations. SAF was defined as the ratio of pixels with positive DAB to those in the tissue mask within a patch. We evaluate the robustness of our pipeline by generating SAF maps at high resolution (16 × 16 μm^2^/patch), which facilitates better identification of high-frequency variations in SAF, and at MRI resolution (0.5 × 0.5 mm^2^/patch), to evaluate the reproducibility of SAF at the resolution used for MRI-SAF analyses.

### 3.2 Evaluation of SAF pipeline

The pipeline was evaluated with two criteria: robustness to artefacts and reproducibility of SAF. These analyses used the evaluation dataset (c.f. Section 2.1.1). We compared pipeline-derived SAF maps with expert-derived (MPG) SAF maps (Pallebage-Gamarallage et al., 2018) that use a manually-derived dataset-specific colour matrix and a stain-specific segmentation threshold. Each manual threshold was calibrated in at least 10 randomly selected, structurally distinct regions.

**Robustness to within-slide artefacts**

To test the pipeline's robustness to within-slide artefacts, we calculated the average column-wise SAF. This horizontal SAF profile was filtered using three bandpass filters (modelled from a Butterworth filter (Butterworth, 1930; Virtanen et al., 2020) applied twice) that are sensitive to different artefacts’ effects: staining gradients (lowpass; < 3 Hz), striping artefact (bandpass; 3–12 Hz) and bandpass plus all other high frequency noise (highpass; > 3 Hz). The frequency bands were qualitatively chosen based on the spatial variability of the artefacts, which may differ between datasets. Within each frequency band, we computed the relative percent change in standard deviation between the manually- and automatically-derived SAF map (**diffstd**) to quantify the reduction in impact from artefacts:

(6) $$diffstd=\frac{std_{manual}-std_{automated}}{std_{manual}}\cdot 100$$

**diffstd** was observed to be sensitive to artefacts that increase the variance of SAF across the slide: striping artefacts increase **diffstd** in the high frequency band, and staining gradients increase **diffstd** in the low frequency band.

**Reproducibility of SAF**

Reproducibility was tested by registering all within-subject slides to the subject's first slide via TIRL (Huszar et al., 2019). We compute an SAF difference map for each co-registered pair of slides (SAF_1,2_) and take the median value across pixels:

(7) $$diffsaf=median\left[\frac{SAF_{1}-SAF_{2}}{(SAF_{1}+SAF_{2})/2}\cdot 100\right]$$

### 3.3 Co-registration of MRI and histology

The pipeline was applied to data from the multiple-region dataset (c.f. Section 2.1.2) with co-registered MRI-histology. The quality of the registration was evaluated according to the alignment of contours representing tissue boundaries and WM/GM contrast. Contours were derived from the PLP SAF maps.

### 3.4 Voxelwise MRI-SAF analyses

MR parameter maps (3D) were resampled into PLP (2D) space for correlation with SAF. As the MR voxels do not align with the 0.5 mm histology grid, the SAF were recalculated for each MR voxel, minimising interpolation effects. Pixels in the SAF maps with values < 5th percentile were identified as non-tissue pixels and discarded. Other outliers were identified using a Huber influence function (tuning coefficient = 2.5), excluding data points with weights < 0.75 (Huber, 2011). We pooled voxelwise data across regions and brains for several linear model analyses between MRI and SAF:

• **Simple correlation**: We correlated each pair of MR (FA, MD, RD, AD, R2*, R1) and SAF (PLP, SMI312, Iba1, CD68) parameters.
• **Partial correlation**: We estimated the unique variance of each MR parameter that is explained by a given stain's SAF, accounting for other stains. Subject ID was used as a covariate to account for between-subject confounds.
• **Multiple linear regression**: We model each MR parameter as a linear combination of all stains (explanatory variables) and the subject ID (confounding variable). We computed each variable's relative importance measure (Grömping, 2015) i.e., the averaged relative contribution of each variable in explaining the variance of the MR parameter after it is added to the model.

As a proof-of-concept, we performed voxelwise analyses on two subjects from the multiple-region dataset (1 x CTL, subject 1; 1 x ALS, subject 2). Analyses were done using WM+GM or WM voxels, where the latter demonstrates sensitivity to subtle microstructural changes rather than gross WM/GM tissue differences

### 3.5 Application to multiple subjects for MRI-PLP

This pipeline can be rapidly applied across many subjects. We demonstrate this by applying the workflow (Sections 3.1, 3.3, 3.4) to 41 PLP slides from ten additional subjects. We correlated MRI with PLP only, as co-registration between other stains and MR data was a work-in-progress. We compared the ten subjects’ output with the two subjects previously analysed. Subject ID was modelled as a covariate.

## 4 Results

### 4.1 Evaluation of SAF pipeline

**Robustness to within-slide artefacts**

**Fig. 3:**
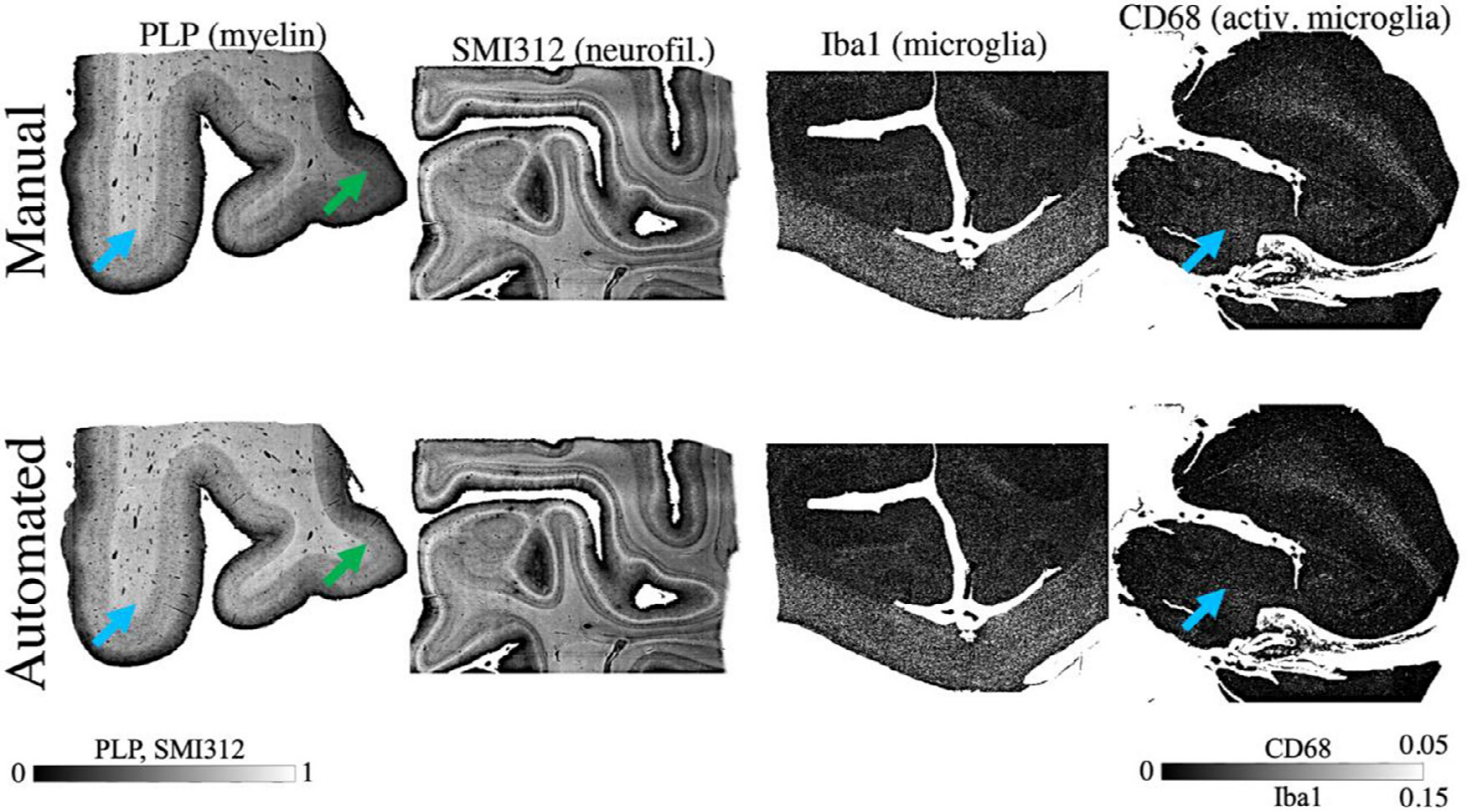
Within-slide artefacts are reduced using the automated pipeline (bottom row). These include noticeable stitching artefacts (blue arrows) and staining gradients (green arrows) originally seen in manually-derived SAF maps (top row). Examples here show PLP slides processed with the pipeline configuration that corrects for staining gradient artefacts (artefact configuration), while SMI312, Iba1 and CD68 slides were processed with the pipeline's default configuration.

The evaluation dataset slides were processed with either the default (CD68, Iba1) or artefact (PLP, SMI312) pipeline. Fig. 3 compares SAF maps from both our automated and manual pipeline. Both configurations of the automated pipeline reduce the impact of stitching artefacts. In the default configuration, this can be attributed to the use of a slide-specific colour matrix, which results in better stain separation compared to the non-specific colour matrices used in manual analyses. The artefact configuration also reduced staining gradient artefacts. In slides with less noticeable artefacts (Iba1), the manual and automated pipelines produce similar results.

**Fig. 4:**
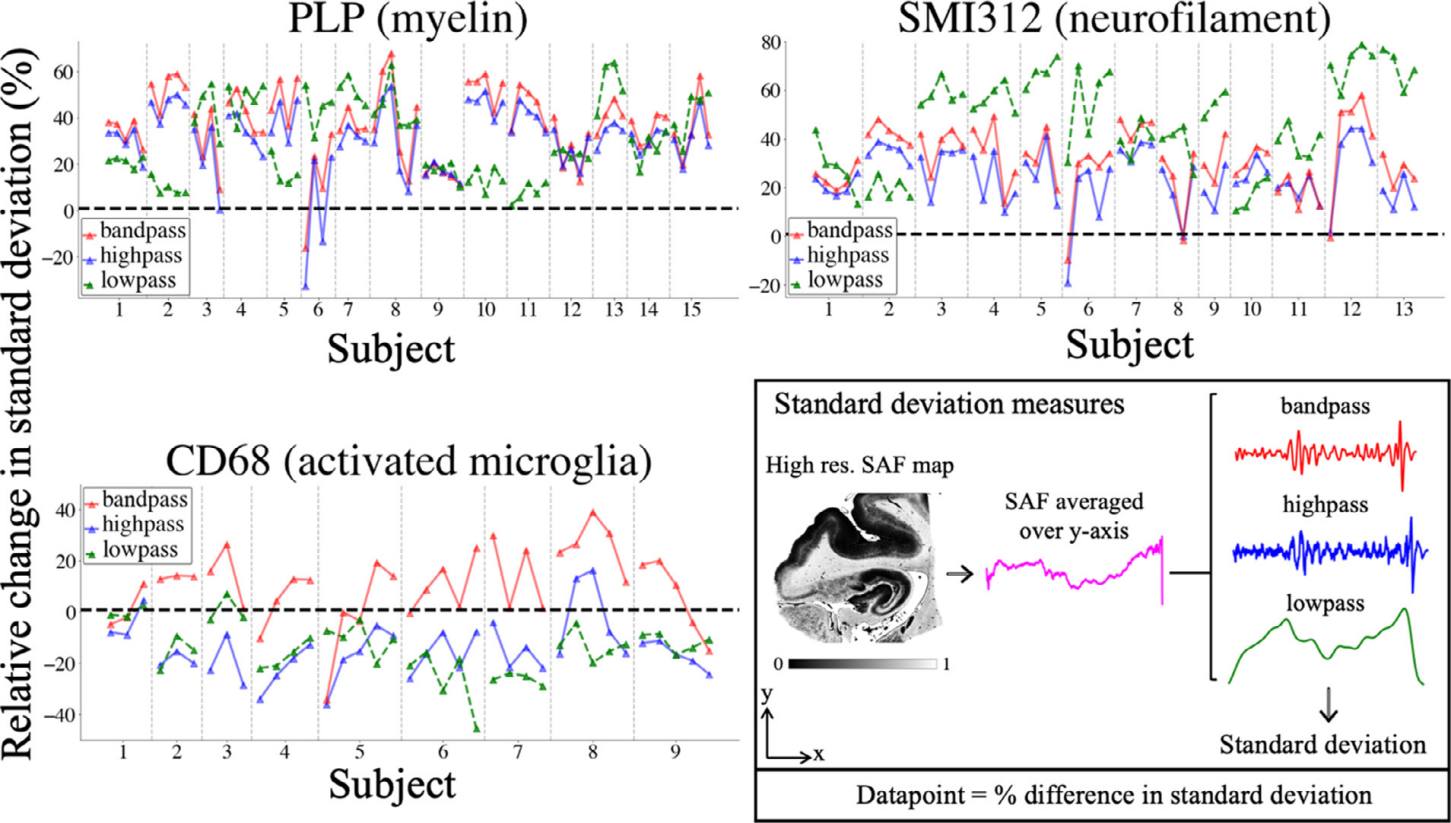
Quantitatively comparing the impact of within-slide artefacts in the manual and automated processing pipelines, as measured using the relative change in standard deviation (100 x (std_manual_ - std _automatic_) / std_manual_). Standard deviation measures (box) are derived by 1) averaging the high-resolution (16 µm/pixel) SAF map along the y-axis, 2) filtering it to produce 3 components (bandpass, highpass and lowpass), and 3) computing the components’ standard deviation. The lowpass filter isolates the staining gradient artefact, whilst the stitching artefact only is captured with the bandpass filter. The highpass filter combines the same striping artefact with all high frequency noise. Each IHC slide is represented as a single datapoint, and slides from the same subject are grouped together and connected by lines. A positive (negative) value indicates that the automatically-derived SAF map is less (more) affected by the associated artefact than the manually-derived SAF map.

In almost all PLP and SMI312 slides (Fig. 4), the **diffstd** for each frequency band (Eq. (6)) was positive, suggesting a reduction in artefacts for the automated pipeline. There was high similarity in highpass and bandpass **diffstd**. This implies the striping artefact's major contribution to overall noise in these slides, and how its impact is especially mitigated with the automated pipeline. The results for CD68 are less conclusive. The automated pipeline reduces the impact of the striping artefact (bandpass **diffstd** is mostly positive) but performs worse than the manual pipeline for the highpass and lowpass filters.

**Reproducibility of SAF**

**Fig. 5:**
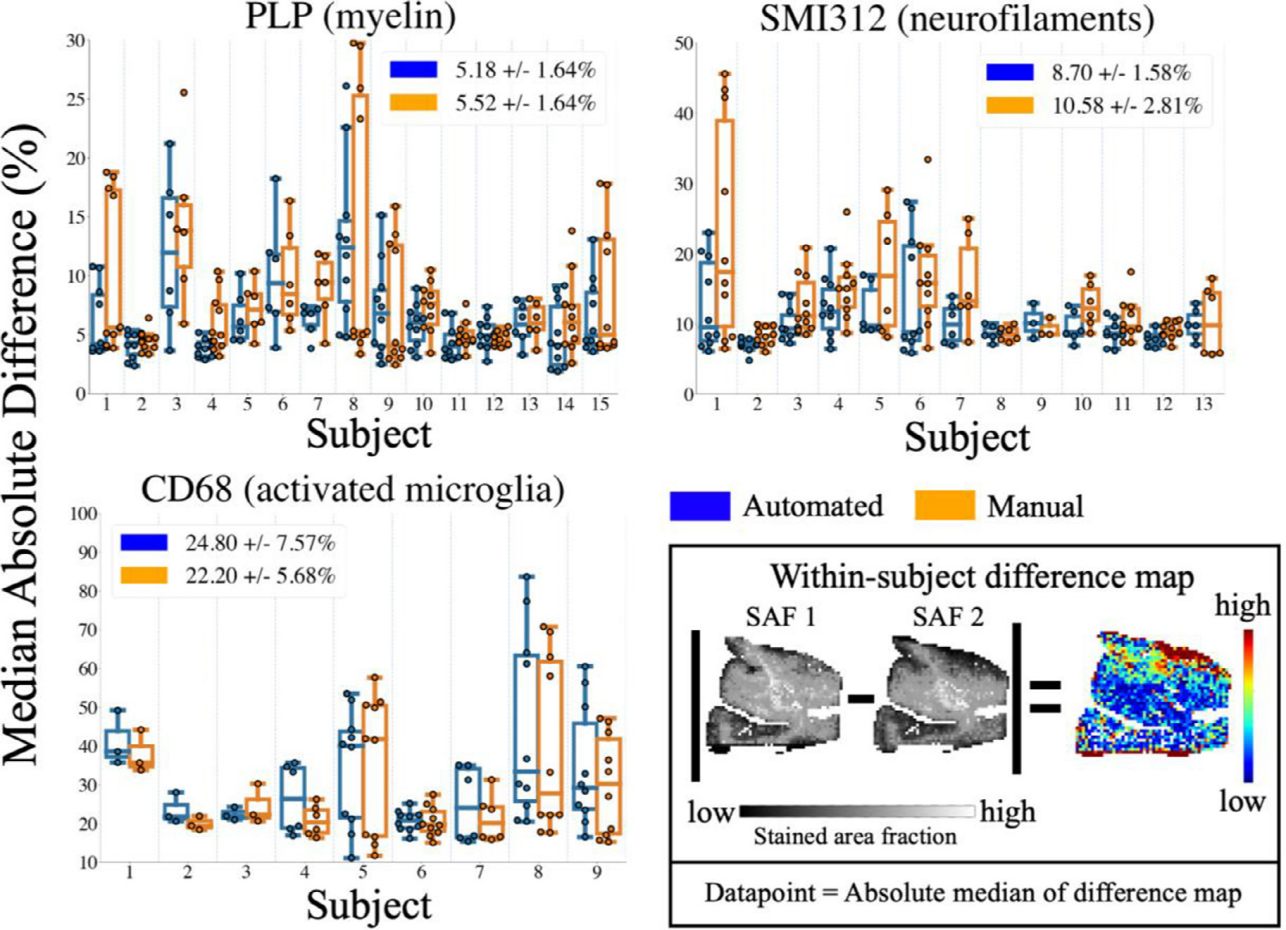
The reproducibility of SAF values across adjacent tissue sections, as measured by the median absolute difference. The median absolute difference (box) was computed by 1) taking the absolute difference of a pair of SAF maps produced from IHC slides extracted from the same region and subject, 2) normalising this difference map by the mean of both SAF maps, and 3) taking the median to represent one datapoint. We used this measure to compare reproducibility of both manually- (orange) and automatically-derived (blue) SAF maps for PLP, SMI312 and CD68. The median and median absolute variance of data points are also shown for each method (legends). Note the change in scale-bar for CD68, where the difference values were generally larger when compared to PLP and SMI312.

Reproducibility of SAF maps (Fig. 5) was quantified with **diffsaf** (Eq. (7)) between within-subject adjacent slides. Manual and automated pipelines were found to have similar reproducibility with a median **diffsaf** of around 5% (PLP), 9% (SMI312) and 20% (CD68). As fewer slides were used when evaluating CD68 (9/15 subjects; 37/73 slides) than in SMI312 (13/15 subjects; 55/72 slides) and PLP (15/15 subjects; 73/73 slides) after manual quality control (c.f. Section 2.1.1), Figs. 4,5 have fewer datapoints.

### 4.2 Co-registration of MRI and histology

**Fig. 6:**
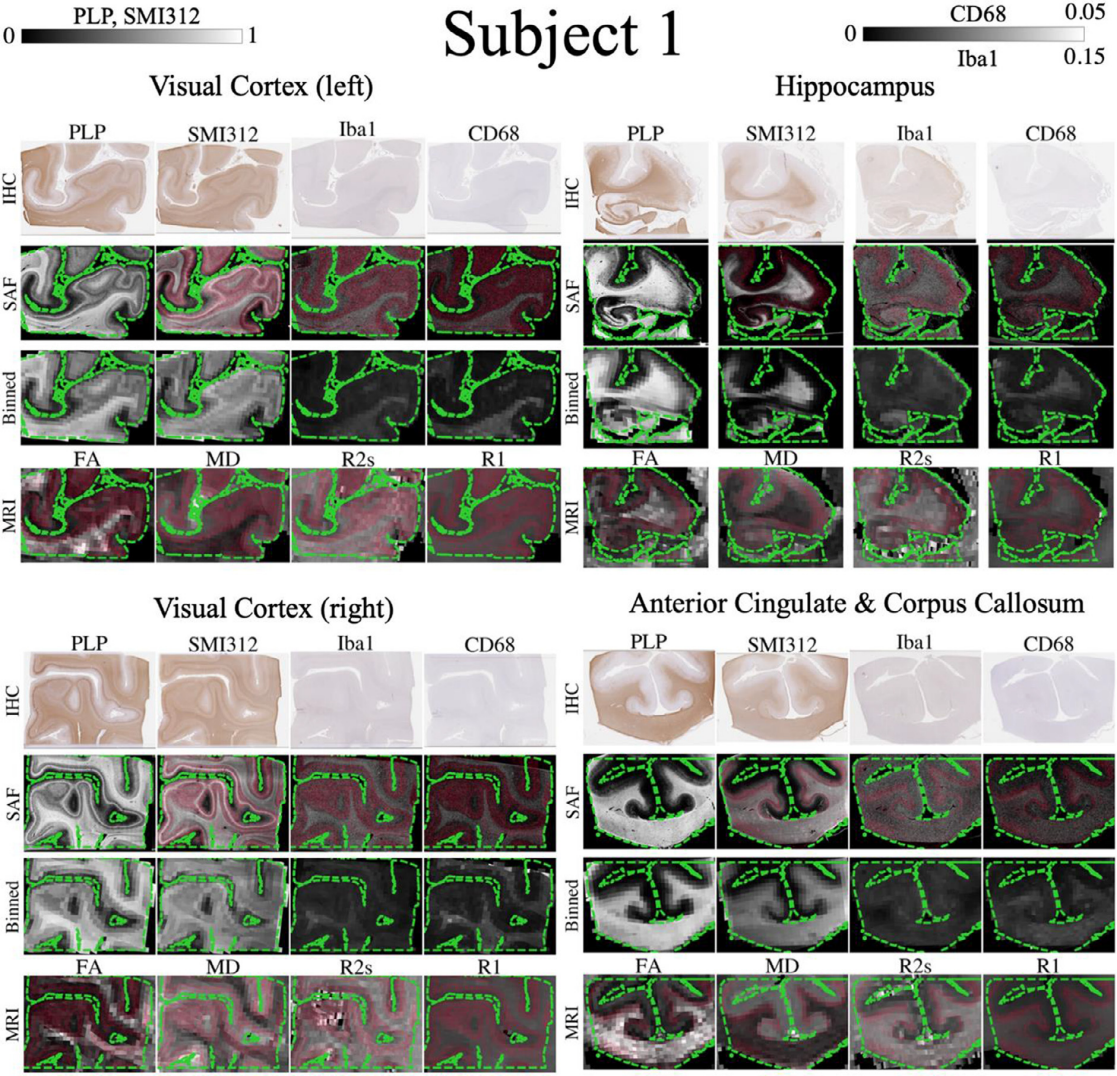
Registration evaluation for all brain regions in subject 1. In each brain region, contours of the tissue mask (green dashed) are overlaid on the co-registered SAF maps at high resolution (first row, 1 pixel here represents SAF calculated in a 16 × 16 μm^2^ patch), SAF maps matching MRI resolution (second row) and MR parameter maps (third row). The white and grey matter interface is shown in red. The tissue boundaries are closely aligned and the high registration accuracy enables us to perform meaningful voxelwise MRI-histology correlations.

Fig. 6 shows good alignment of the tissue mask (green contour) and WM/GM interface (red contour) for subject 1. Subject 2 had similar results (Figure S4). Of the remaining subjects, 10 had good quality registrations (Figure S5) and 3 were excluded from subsequent analysis due to substantial misalignment. Additional quantitative evaluation of the dataset registration can be found in (Huszar et al., 2019).

### 4.3 Voxelwise MRI-SAF analyses

Eight IHC slides from different regions (Figs. 6, S5) in subjects 1 and 2 were mapped onto MRI data. For each MR parameter, up to 3% of voxels were classified as outliers and removed.

#### 4.3.1 Simple correlation

**Fig. 7:**
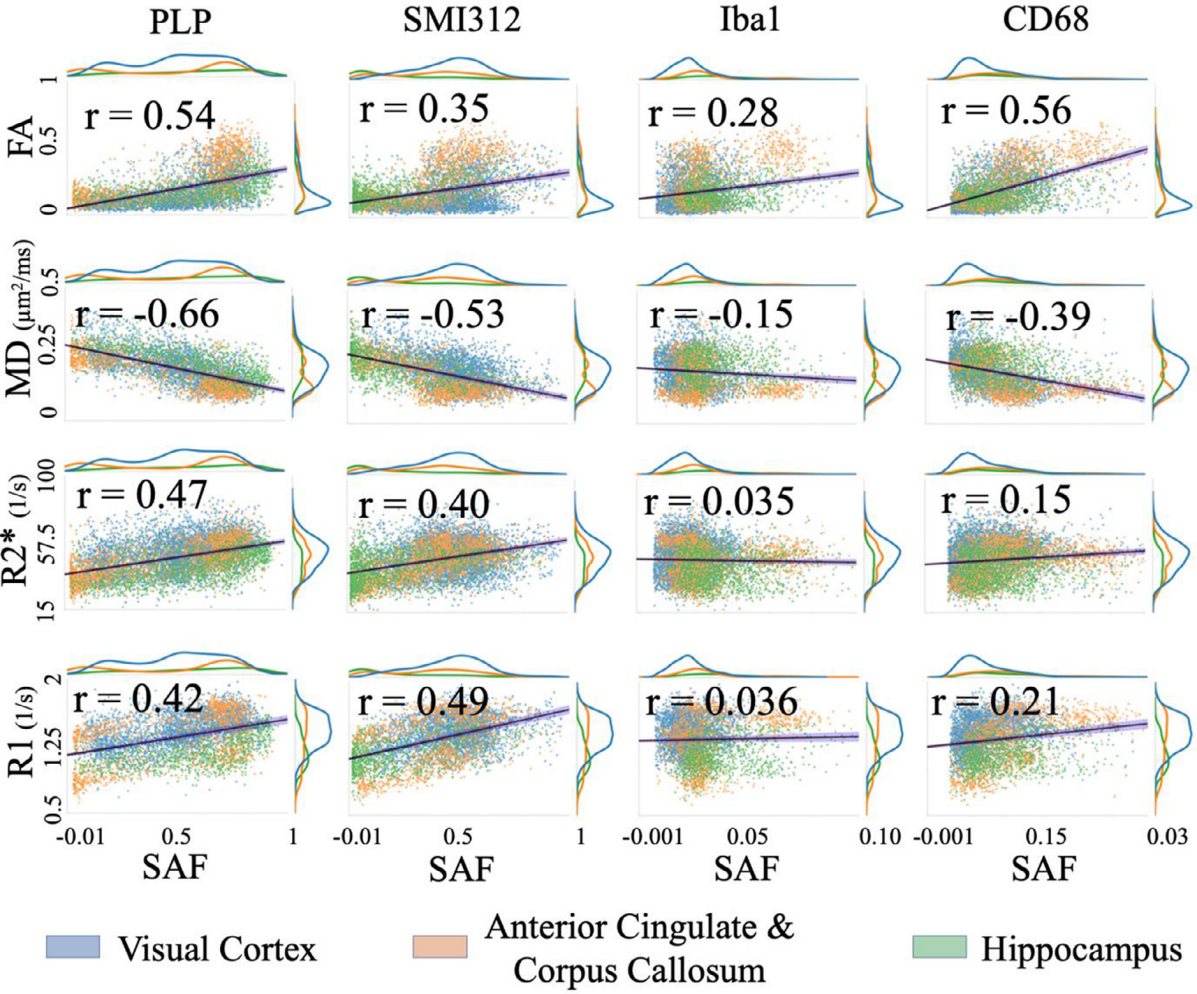
Correlating MR parameters (DTI FA, MD, R2* and R1) with IHC SAF (WM+GM). The line of best fit (black line) and corresponding Pearson correlation coefficients, r, are overlaid. Confidence intervals (99%) are shown in blue shade. The visual cortex (blue), anterior cingulate (orange) and hippocampus (green) provide good dynamic range for the MR parameters and SAFs.

Simple correlation was used to relate each MRI-SAF pair (Fig. 7). While correlations with Iba1 appear low (|r| = 0.035–0.28), CD68 correlated well with FA (*r* = 0.56) and MD (*r* = −0.39). The scatterplots also suggest nonlinear trends for FA with PLP and SMI312. Similar analysis of WM only is shown in Figure S6, where FA shows a strong correlation with CD68 (*r* = 0.33) and R2* with Iba1 (*r* = −0.30). Scatterplots with AD and RD are shown in Figure S7. In WM+GM, AD and RD correlated negatively with all stains. When analysing the WM only, notable results include the correlation of AD with Iba1 (*r* = 0.27) and RD with CD68 (*r* = −0.21).

#### 4.3.2 Partial correlation

**Fig. 8:**
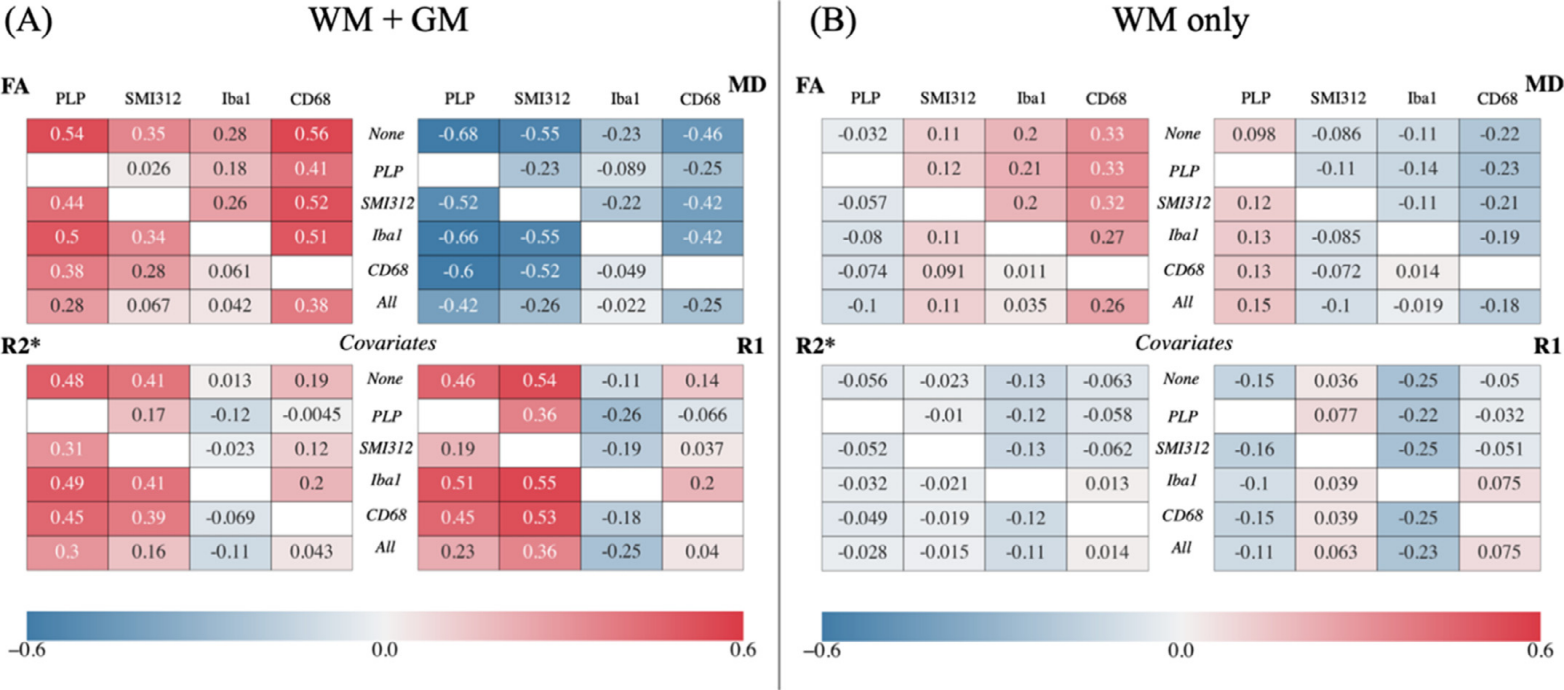
Partial correlation analysis between MR parameters (FA, MD, R2* and R1) and IHC stains for (A) both WM+GM voxels and (B) for WM only. In each quadrant for either (A) or (B), the top row (“None”) gives the correlation coefficients when accounting for subject ID only. The middle rows give the partial correlation coefficient controlling for one of the other stains and subject ID (italicised labels in the second to fifth rows). The bottom row (“All”) gives the partial correlation coefficient controlling for all stains and subject ID. Note that correlations between FA/MD with CD68 remain relatively high, even after accounting for all other stains. *PLP (myelin); SMI312 (neurofilaments); Iba1 (microglia); CD68 (activated microglia).*

For each stain, correlations were calculated after regressing out one other stain or all other stains as covariates. We show correlation coefficients when including all voxels (WM+GM) (Fig. 8A), or WM only (Fig. 8B). Similar data for AD and RD are shown in Figure S8.

**WM and GM**

When accounting for all other stains (Figs. 8A and S8A, bottom rows), FA is best explained by CD68 (*r* = 0.38), MD/AD/RD/R2* by PLP (*r* = −0.42/*r* = −0.65/*r* = −0.68/*r* = 0.30) and R1 by SMI312 (*r* = 0.36). We observed two main results: the shared explained variance between PLP (myelin) and SMI312 (neurofilaments), and between Iba1 (all microglia) and CD68 (activated microglia).

First, the correlation coefficient between FA and SMI312 is greatly reduced after accounting for PLP (*r* = 0.35 to 0.026), whilst the correlation coefficient between FA and PLP is marginally reduced after accounting for SMI312 (*r* = 0.54 to 0.44). Similar effects are observed between MD and PLP/SMI312, and between R2* and PLP/SMI312. The opposite effect is seen in R1: the correlation coefficient between R1 and PLP decreases significantly when covarying for SMI312 (*r* = 0.46 to 0.19), but the correlation coefficient between R1 and SMI312 is marginally reduced when accounting for PLP (*r* = 0.54 to 0.36). Second, we saw that the high correlations of FA/RD with CD68 marginally decrease after accounting for Iba1 (r_FA_ = 0.56 to 0.5; r_RD_ = −0.50 to −0.45). Conversely, the apparent correlations with Iba1 are minimal after accounting for CD68 (r_FA_ = 0.28 to 0.061; r_RD_ = −0.24 to −0.029). We note similar trends for correlations of MD with CD68/Iba1.

**WM only**

Figs. 8B and S8B show the same analysis for voxels found in the WM only. When accounting for all other stains, FA/MD/RD are best explained by CD68 (*r* = 0.26/*r* = −0.18/*r* = −0.22), AD by PLP (*r* = 0.10), R2*/R1 by Iba1 (*r* = −0.11/ *r* = −0.23). When compared to our previous results for WM+GM voxels, the correlation coefficient between most MR parameters and PLP/SMI312 are greatly reduced and/or close to zero. This behaviour is not seen for CD68 and Iba1. In WM, CD68 explains the most unique variance in FA and RD. Notably, these correlations remain relatively unchanged when accounting for other stains (r_FA_ = 0.33 to 0.26; r_RD_ = −0.29 to −0.22). Similar behaviour is observed when relating CD68 with MD, and Iba1 with R1/R2*.

#### 4.3.3 Multiple linear regression

We show the regression and fitted correlation coefficients (i.e., square root of the coefficient of determination) when including voxels from WM+GM, or WM only (Tables 1A, S2A). Using multiple stains better explains the variance in MR parameters than individual stains. We also show the relative importance of each predictor (Tables 1B, S2B). The subject predictor had the highest relative importance in predicting R1 in all voxels, and MD/R2*/R1 in WM voxels only. This implies that between-subject confounds substantially influence MR parameters.

### 4.4 Application to multiple subjects for MRI-PLP

Similar regression coefficients were seen for the larger group (*n* = 10, Figure S9 right) compared to the original analysis (*n* = 2, Figure S9 left), with the estimated effect (**β**) being highly consistent for FA, MD and R2*, but less consistent for R1.

## 5 Discussion

To facilitate high-throughput MRI-histology analyses, we introduce an automated pipeline to extract stain area fraction (SAF) from immunohistochemical (IHC) stains. Using high-quality co-registration, we performed whole-slide voxelwise MRI-SAF comparisons. The pipeline was applied to post-mortem human brain data from multiple subjects, relating slides stained for myelin (PLP), neurofilaments (SMI312), microglia (Iba1) and activated microglia (CD68) to MR parameters (FA, MD, RD, AD, R2*, R1).

Our approach has three advantages over previous studies. First, while most MRI-SAF studies use manually-derived SAF, our pipeline deploys data-driven algorithms (Geijs et al., 2018; Yuan et al., 2015). This enables the pipeline to be applied rapidly to larger datasets without expert intervention. Second, our pipeline can handle key artefacts (non-biological sources of variation) which impact SAF maps. This is an improvement over previous automated pipelines (Grussu et al., 2017; Khodanovich et al., 2019; Praet et al., 2018). Third, the pipeline is generalisable to multiple IHC stains, allowing for analysis spanning multiple microstructural sources. Combined with recent advances in MRI-histology co-registration (Huszar et al., 2019), the pipeline may enable standardised analyses for voxelwise MRI-SAF comparisons (Lazari and Lipp, 2021).

The pipeline facilitates voxelwise MRI-SAF analyses that inspire more confidence than ROI-based analyses, which are biased by the choice of ROI and/or dilute localised effects-of-interest. Further, most studies perform simple correlations with only a single or a few histological markers (Yano et al., 2018; Seewann et al., 2009; Peters et al., 2019). We extend this analysis to partial correlation and multiple linear regression relating multimodal MRI to multiple IHC stains. This presents two key benefits. First, partial correlations can demonstrate sensitivity to specific microstructural changes such as microglial activation (changes in CD68 but not Iba1), demyelination (changes in PLP but not SMI312) and axonal loss (simultaneous changes in SMI312 and PLP). This may aid with disease diagnosis and/or staging. Here, our pipeline is applied to a dataset that was specifically acquired for future investigation into ALS neuropathology, as motivated in our previous publication that presented the study design (Pallebage-Gamarallage et al., 2018). Currently, we refrain from making any strong conclusions with respect to ALS pathology since our MRI-SAF results are from only two subjects, where co-registration for other subjects is still a work-in-progress. However, future work will draw on MRI-SAF results from the entire multi-subject dataset to provide an in-depth evaluation of the cellular bases of MRI in the context of ALS neuropathology. Second, both the partial correlation and multiple linear regression analyses can account for confound variables, which may otherwise influence our results. Subject-specific confounds contributed to significant variation in R1/MD/R2* (relative importance in Table 1B). This may be due to different post-mortem intervals (3 versus 2 days) and/or fixation times (45 versus 139 days), both of which are known to influence MR parameters (R1, R2*: (Shatil et al., 2018; McNab et al., 2009; Foxley et al., 2014); MD: (McNab et al., 2009; Foxley et al., 2014)). Notably, FA-microstructure mappings were independent of subject confounds, given that post-mortem tissue exhibits similar patterns of FA when pooling voxels from WM+GM (Sun et al., 2005; Sun et al., 2003; Roebroeck et al., 2019). A more in-depth analysis of these confounds (e.g., whether post-mortem interval or fixation time is the primary driver of between-subject variance) is left for future work within the larger multiple-subject dataset.

**Table 1:** Multiple linear regression predicting MR parameters using multiple IHC stains. Values are computed from WM+GM, or from WM only. A “subject” variable was also included to account for confounds such as post-mortem interval and age effects. As the predictors are unitless, all offsets and regression slopes are given in units of MR parameters. A: The regression coefficients and correlation coefficients r_fit_. B: The relative importance of each stain describes the amount of variance it can explain in an MR parameter, averaged over all permutations of multiple regressions that include the specific stain. Values are normalised across stains to get a unit percentage.

All voxels/WM only							
(A) Multiple regression offset and slopes							
	Offset	PLP	SMI312	Iba1	CD68	Subject	r_fit_
**FA**	−0.00142*/0.255	0.173/−0.173	0.0429/0.144	0.252/0.410*	10.5/8.81	−0.0112/−0.0247	0.645/0.353
**MD [µm**^**2**^**/ms]**	0.286/0.118	−0.112/0.0610	−0.070/−0.0323	−0.0629*/−0.0531*	−2.55/−1.40	0.0366/0.0310	0.745/0.429
R2* [1/s]	39.2/61.2	16.5/−2.22*	9.04/−0.889*	−61.2/−55.2	80.7/19.6*	−3.73/−5.54	0.536/0.455
**R1 [1/s]**	1.04/1.52	0.220/−0.217	0.382/0.0878	−2.48/−2.71	1.34/1.89	0.234/0.268	0.710/0.637

(B) Relative Importance [%]					
	PLP	SMI312	Iba1	CD68	Subject
**FA**	36.0/4.32	11.9/8.75	7.04/15.1	44.5/69.2	0.55/2.66
**MD**	45.0/6.43	25.7/2.40	1.69/4.99	14.5/16.2	13.1/70.0
R2*	51.3/0.47	30.1/1.97	2.90/22.6	3.86/3.62	11.8/71.3
**R1**	18.7/4.54	30.6/2.39	3.15/5.88	2.93/0.443	44.6/86.8

*not significant (*p*>0.05)

### 5.1 Evaluation of SAF pipeline

Our results show the pipeline's robustness to common IHC artefacts, while maintaining similar reproducibility to manually-derived SAF values (Fig. 3, Fig. 4, Fig. 5). This is especially in PLP slides, which typically displayed within-slide artefacts. Conversely, CD68 appears less reproducible for both manual and automated processing. CD68 stains sparse, small, activated microglia (soma diameter∼10 µm (Kozlowski and Weimer, 2012)). Here, the **diffstd** metric may be unsuitable as the assumption of low biological variability across slides may not be met. Inspection of the CD68 segmentation revealed that the automated pipeline correctly segmented more activated microglia relative to the manual method. The inclusion of more sparsely distributed microglia may add biological variability to the SAF, resulting in higher low- and highpass **diffstd**. Staining gradients were not visibly present in CD68 slides, though some striping was visible on the manually-derived SAF maps. The automated pipeline reduced this striping, as reflected in the improved bandpass **diffstd**.

### 5.2 Voxelwise MRI-SAF analyses

Our correlation results agree with previous studies for myelin (Lazari and Lipp, 2021; Mancini et al., 2020; Seewann et al., 2009; Bagnato et al., 2018) and neurofilaments (Grussu et al., 2017). Activated microglia correlates positively with FA and negatively with MD/RD, similar to that reported in the human spinal cord (Grussu et al., 2017).

#### 5.2.1 Myelin and neurofilaments

Our partial correlations for PLP (myelin) and SMI312 (neurofilaments) demonstrate pitfalls in performing pairwise MRI-histology correlations without controlling for other tissue features. Our simple correlations from WM+GM voxels reveal an apparent relationship between neurofilaments and FA, MD, R2*; however, our partial correlation results that account for myelin find no such relationship. Thus, one may erroneously conclude from simple correlations that anisotropy is affected by myelin and neurofilament (axons, dendrites) load, whereas our partial correlations show that anisotropy is primarily related to myelin density, with no unique variance attributed to dendritic load. As we do not account for microstructure orientation dispersion—where the orientational coherence of axons and dendrites may drive variations in diffusion anisotropy (Beaulieu, 2002)—we cannot determine whether the FA-PLP correlation reflects a direct relationship with myelin density or an indirect relationship with microstructure dispersion. These results imply that the portion of microstructure dispersion that affects FA is unrelated to dendritic load; if it were, we expect a high correlation between FA and dendritic load, with microstructure dispersion being the main confound in driving this relationship. This is something we hope to investigate further in the future. MD and R2* are similarly affected by myelin load, though some specificity to unmyelinated axons and/or dendrites remains.

Conversely, SMI312 explains unique variance in R1, even after accounting for PLP. This implies that R1 is sensitive to neurites in general, rather than myelin alone. This is noteworthy, given the studies correlating myelin with R1 (Stüber et al., 2014; Hakkarainen et al., 2016; Warntjes et al., 2017) without considering the impact of unmyelinated axons and/or dendrites.

Finally, in WM only, all PLP/SMI312 associations are reduced, suggesting WM/GM contrast as the primary driver. Taken together, our results may explain the wide range of MRI-myelin correlation coefficients reported in two reviews (Figs. 4, 5 in (Lazari and Lipp, 2021); Fig. 4 in (Mancini et al., 2020)).

#### 5.2.2 Microglia

Our results suggest a relationship between CD68 (activated microglia, a biomarker for neuroinflammation (Bachiller et al., 2018; Geloso et al., 2017)) and FA/MD/RD, after accounting for all other stains (e.g., demyelination, axonal loss or general microglia infiltration). This relationship is present when considering all voxels (WM+GM) and WM only, demonstrating MR sensitivity to subtle microstructural changes rather than gross WM/GM differences. This result may be linked to microstructural changes in response to neuroinflammation that are unaccounted for in our histology metrics, or non-trivial changes in “activated” versus “non-activated” microglia morphology. For example, activated microglia often have more retracted, thicker processes and different soma sizes and shapes compared to their non-activated counterparts (Davis et al., 2017). This may contribute to increased restriction and/or hinderance of diffusion in the extra-axonal space, leading to an observed reduction in RD and a resultant increase in diffusion anisotropy (FA). While some report decreased FA in diseases associated with neuroinflammation (Chiang et al., 2017; Samara et al., 2020), they do not measure microglial activation and generally attribute the changes in FA to WM damage. Future research into measuring microglial activation using MRI will benefit from the development of advanced biophysical models (Taquet et al., 2019; Garcia-Hernandez et al., 2022; Zhang et al., 2012; Rafipoor et al., 2022) that can simultaneously account for multiple changes in the microstructure (demyelination, axonal damage, microglial infiltration, exchange effects etc.) and provide superior specificity to the DTI metrics.

Activated microglia is known to colocalise with iron (Kwan et al., 2012; Kaunzner et al., 2019) and correlate positively with R1/R2* (Wiggermann et al., 2017; Hametner et al., 2018; Roebroeck et al., 2019; Langkammer et al., 2010). However, CD68 is found to weakly correlate with R1/R2* after accounting for subject-specific confounds (Fig. 8, Table 1). Our results also suggest a negative correlation between non-activated microglia (Iba1 after accounting for CD68) and R1/R2*. Future studies may incorporate iron-stained IHC to elucidate the contributions of iron to relaxometry measures.

### 5.3 Limitations

There are limitations to this study. First, we use SAF—a semi-quantitative metric defined via a segmentation threshold—to quantify MRI-histology relationships. Depending on how the SAF threshold is derived, this may result in subtly different SAF maps. Further, SAF may not scale linearly with protein density, as anything above the stain threshold is considered positive stain. While our results show key trends between MR parameters and proteins, the exact MRI-SAF slopes may not be directly comparable to other studies using different thresholding methods or stain densities. Here, a key advantage of our data-driven SAF is that it is less sensitive to non-biological staining variations both within and between slides, which if unaccounted for, can bias stain density metrics. Third, we do not incorporate other histological metrics that may relate to MRI. For example, we do not include the fibre orientation dispersion and microglial cell morphology, which may explain variance in FA or other MR parameters. Finally, our pipeline does not address smaller-scale within-slide artefacts, such as artefactual staining of vasculature and folding artefacts at tissue edges (Suvarna et al., 2019). These might bias estimated SAF. Since our analysis occurs at the slide-level, manually excluding all smaller-scale artefacts would be very time-intensive. Following a qualitative check of the slides, we observed the staining of vasculature and folding artefacts to be relatively sparse, with a tendency to appear on the tissue edges. We do not anticipate the effect size of these artefacts to be significant.

## 6 Conclusion

We introduce an automated pipeline that extracts SAF maps from IHC slides. By design, the pipeline is generalisable to multiple IHC stains and found to be robust to artefacts from tissue staining and/or slide digitisation. Pipeline-derived SAF values were found to be as reproducible as expert-derived SAF estimates. The pipeline was applied to co-registered MRI and IHC data from post-mortem human brains, where we perform multiple linear analyses (simple correlation, partial correlation and multiple linear regression) to investigate voxelwise relationships between MRI- and IHC-derived parameters. Our results emphasise the need to simultaneously analyse multiple stains when validating MRI, as to avoid misleading inference due to the spatial covariance of multiple microstructural features. Interestingly, we found several diffusion-weighted metrics’ sensitivity to activated microglia–a biomarker of neuroinflammation. This result held after accounting for spatial covariance of other stains (myelin, neurofilaments and non-activated microglia), suggesting MR specificity to aspects of neuroinflammation related to microglial activation, irrespective of other microstructural changes (axonal loss, demyelination or general microglia infiltration). Finally, subject ID was found to be the strongest predictor of some MR parameters, highlighting the need to consider tissue processing confounds when comparing post-mortem MRI across subjects. Together, our results demonstrate the pipeline as a valuable tool for IHC analyses and future investigations relating MRI to disease pathology.

## Authors’ contributions

**DZLK** conceived the study design, developed the histology processing pipeline, implemented the MRI-histology workflow, conducted data analyses, and drafted the manuscript. **SJ** conceived the study design, contributed to the design of the MRI-histology workflow and data analyses. **INH** developed the TIRL registration platform, performed the MRI-histology co-registration, designed the acquisition of the reproducibility dataset, and advised on data analyses. **JM** contributed to the histology processing pipeline. **BCT** analysed the diffusion MRI and T1 data. **SF** developed the MRI protocols and acquired the MRI data. **CW** analysed the R2* data. **CS** contributed to the acquisition of the multiple-region dataset. **AS** performed the histological processing, curated histological data, and provided expert feedback on histology analysis. **OA** designed the brain sampling strategy and provided expert feedback on histological analysis. **MPG** optimised histological protocols, designed the brain sampling strategy, performed histology analysis, evaluated histological segmentations, and provided expert feedback on histological analysis. **KLM** conceived the study design, designed the post-mortem MRI protocol, contributed to the MRI-histology workflow, contributed to all data analysis, and helped draft the manuscript. **AFDH** conceived the study design, contributed to the MRI-histology workflow, contributed to all data analysis, and helped draft the manuscript.

## Declaration of Competing Interest

None.

## Data Availability

The code for this work is publicly available at https://git.fmrib.ox.ac.uk/spet4877/ihcpy. The data will soon be available on https://open.win.ox.ac.uk/DigitalBrainBank/.

## Appendix Supplementary materials

Image, application 1
