# Supplementary material for: An automated pipeline for extracting histological stain area fraction for voxelwise quantitative MRI-histology comparisons
Source: Neuroimage. 2022 Dec 1;264:119726. doi: 10.1016/j.neuroimage.2022.119726 (PMC10933753; doi:10.1016/j.neuroimage.2022.119726)
Supplement: Supplementary file 1 [file mmc1.docx]

## Supplementary Materials

### Appendix A. Colour Channel Separation

The Beer-Lambert’s law relates the attenuation of light through a material (I_1_ = I_0_ * 10^-A^), to the concentration (C) and absorptivity, or attenuation coefficient, ε of the medium. This expression can be used to calculate the stain density, where the absorbance A is defined as:


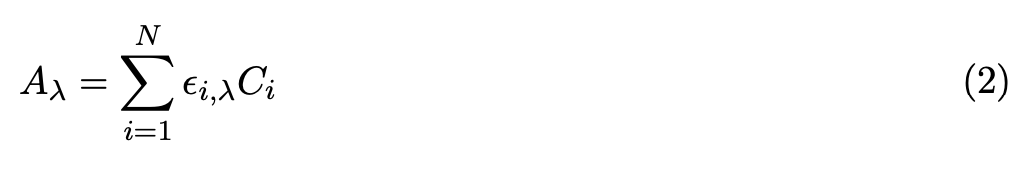


*N* is the number of stains, ε_i,λ_ denotes the attenuation coefficient of i^th^ stain for light at wavelength λ, and C_i_ represents the stain density of i^th^ stain. For λ corresponding to red, green and blue colour channels, the stain density (**Cstain**) of DAB, hematoxylin, and the leftover staining (residual) for a pixel with Idet,λ on the slide can be derived as:


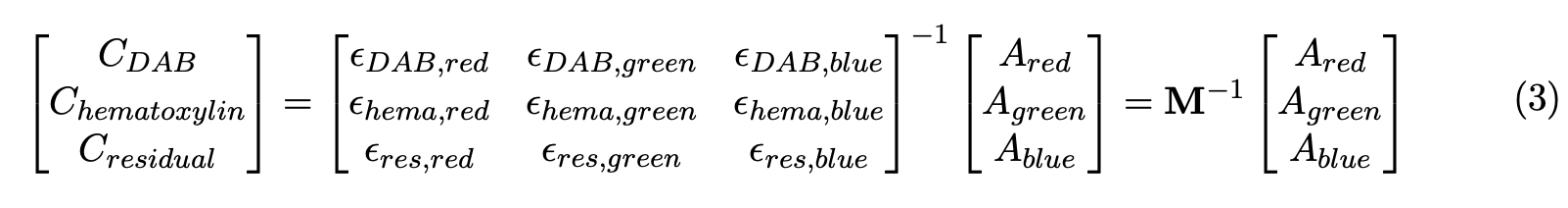


Estimation of stain density, **Cstain**, is a least-squares error minimization problem. However, the rows of M (i.e. the stain-specific colour vectors) are not constrained to be orthogonal such that **Cstain** can be negative. While this is mathematically valid, it leads to a problematic interpretation of Cstain as physical stain concentration.

We address this by using an approach designed to emulate the non-negative least squares (NNLS) algorithm. We call this the pseudo-NNLS (pNNLS). The pNNLS is used over NNLS as it computationally much faster than the NNLS. The pNNLS first estimates a stain’s density Cstain via a standard matrix inversion. In the non-physical case where a stain’s Cstain is negative, the stain’s density value is set to 0. The other stain’s positive density value is then re-computed by projecting the **AR,G,B** to the specific stain’s colour vector. We conducted simulations (not shown) to confirm that stain densities derived from both pNNLS and NNLS are effectively identical. Note that since all AR,G,B values are strictly positive, the case of having negative densities for both stains simultaneously is not possible.

### Appendix B. Figures and Tables

| Brains | CTL 1-3, ALS 1 | ALS 2, 3 | ALS 4,5 | ALS 6,7 | ALS 8, 9 | ALS 10 | ALS 11, 12, 13 |
| --- | --- | --- | --- | --- | --- | --- | --- |
| No. inversion times | 6 | 6 | 6 | 6 | 6 | 6 | 6 |
| TI (ms) | 30, 60, 120, 240,480,  935 | 30, 60, 120, 240, 480, 945 | 30, 60, 120, 240, 480,  945 | 30, 60, 120, 240, 480,  945 | 30, 60, 120, 240, 480, 945 | 30, 60, 120, 240, 480, 940 | 30, 60, 120, 240, 480, 960 |
| TE (ms) | 14.2 | 11.5 | 11.5 | 11.5 | 11.5 | 12.5 | 8.14 |
| TR (ms) | 1000 | 1000 | 1000 | 1000 | 1000 | 1000 | 1000 |
|  |  |  |  |  |  |  |  |
| resolution (mm^3^) | 0.90 x 0.90 x 0.90 | 0.80 x 0.80 x 1.60 | 0.80 x 0.80  x 1.60 | 0.80 x 0.80 x 1.60 | 0.80 x 0.80 x 1.60 | 0.65 x 0.65 x 1.30 | 1.0 x 1.0 x 1.20 |
| Flip Angle (degrees) | 180 | 180 | 180 | 180 | 180 | 180 | 180 |
| Bandwidth (Hz/pixel) | 130 | 195 | 195 | 195 | 195 | 170 | 318 |
| Turbo factor | 4 | 4 | 4 | 4 | 4 | 4 | 4 |
| Slice resolution (%) | 100% | 100% | 100% | 50% | 50% | 50% | 100% |
| Remarks | First two inversion time volumes not used in T1 map estimation |  | Final inversion time volume not used in T1 map estimation | Final inversion time volume not used in T1 map estimation |  | Final inversion time volume not used in T1 map estimation |  |

**Table S1**: Acquisition for parameter multi-TI turbo spin-echo protocol use for estimating R1 maps. Note data from CTL 1 (subject 1) and ALS 1 (subject 2) were used to obtain MRI-SAF results relating multiple SAFs with multiple MR parameters.


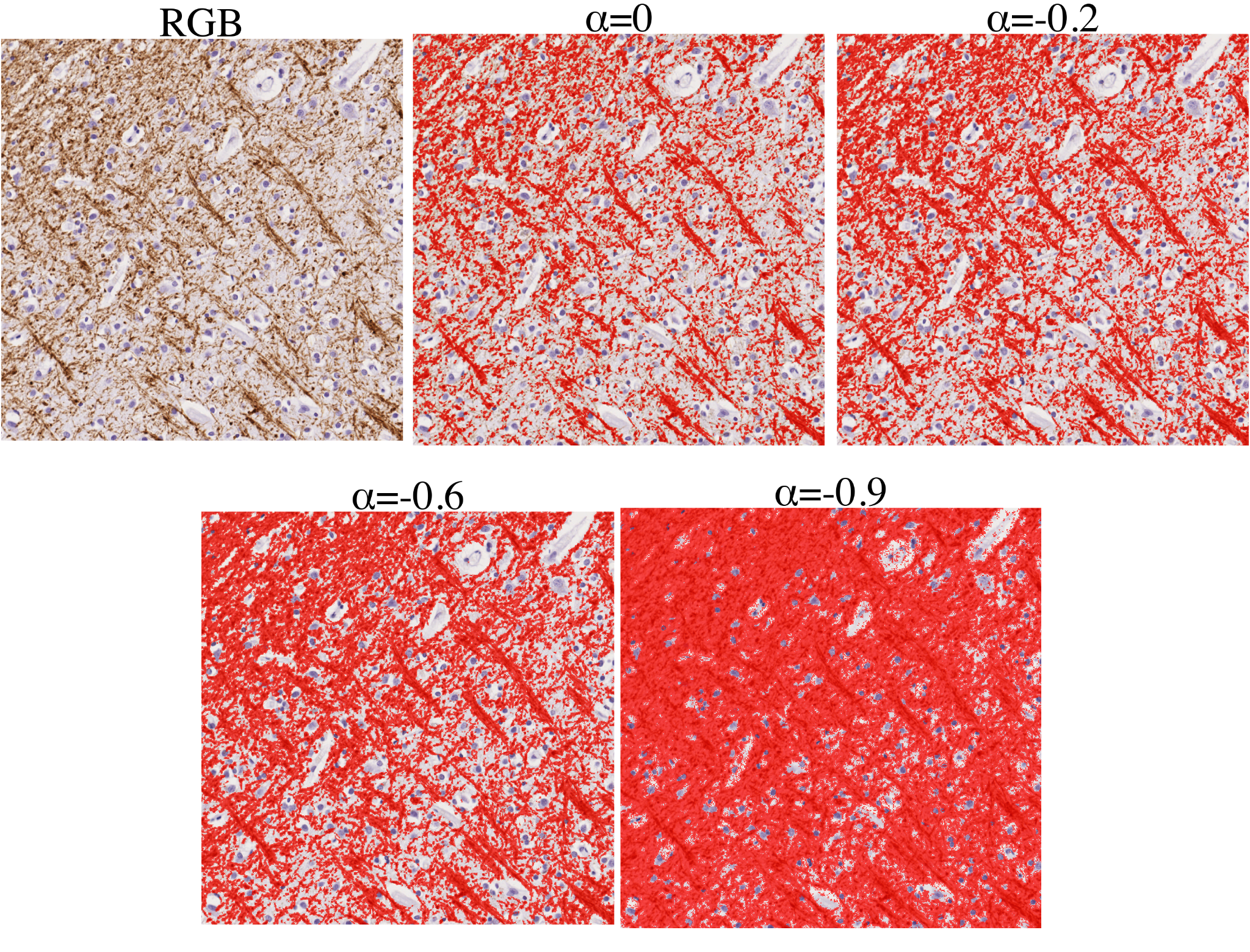


**Figure S1**: Optimising hyperparameter **α** to produce an accurate segmentation of the target protein. We show this in an example myelin-stained (PLP) patch. The hyperparameter α affects the segmentation of the protein-of-interest. Here, varying **α** from 0 to -0.9 slowly increases the number of pixels we identify as protein-of-interest. Based on expert input, we identified **α**=-0.6 as optimal for accurately segmenting myelin. Once **α** is set for a single stain, we apply it to all slides of that stain.


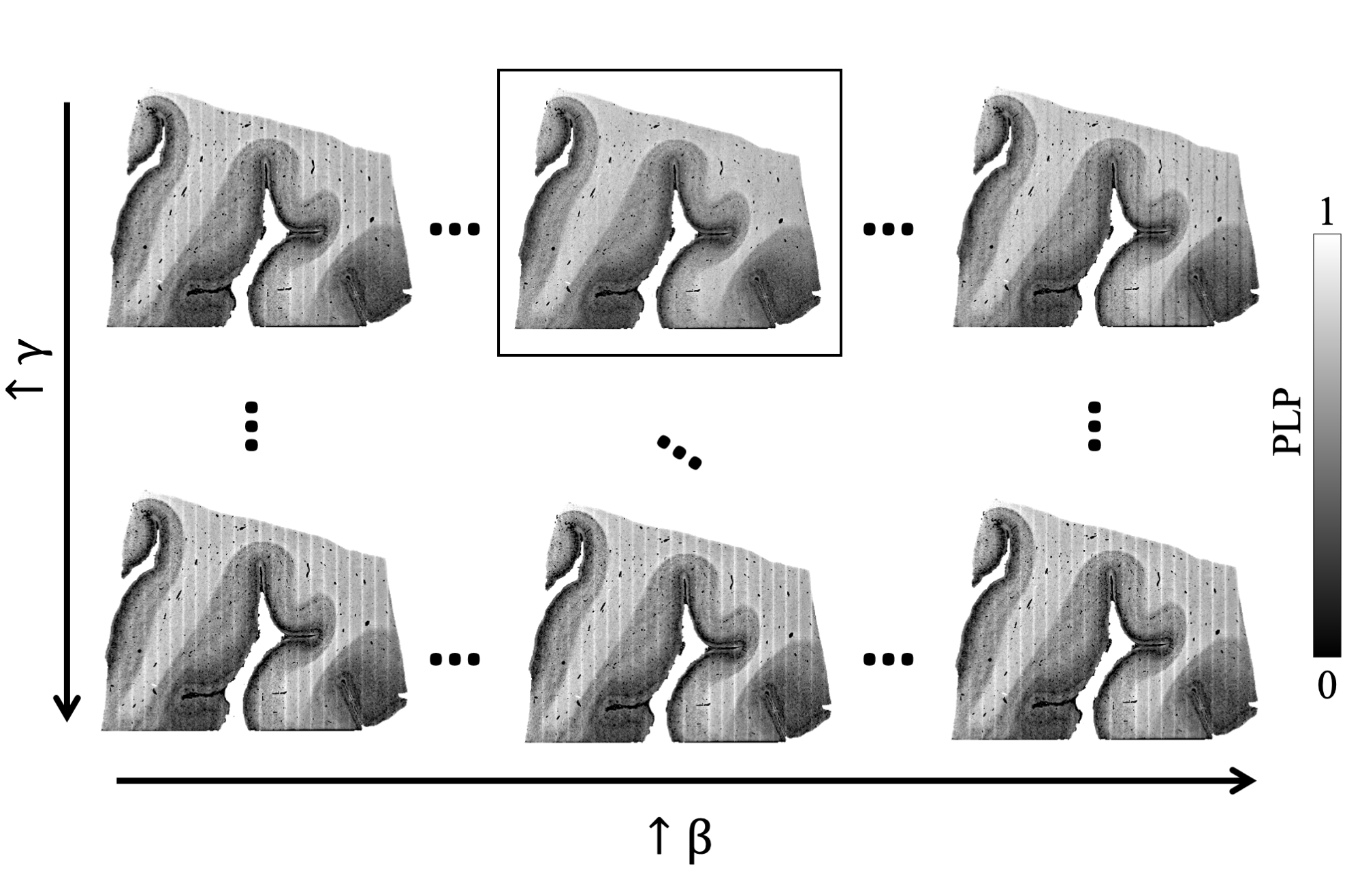


**Figure S2**: Optimising hyperparameters **β**, **γ** to produce a resultant stain area fraction map. These hyperparameters directly address the striping artefacts, and are optimised for for each slide using grid search. In this example myelin-stained (PLP) slide, we see substantial striping in the SAF map (top left; **β**=0; **γ**=0), An increase in **β** is needed to produce segmentation threshold that compensate for changes due to striping present (black square; **β**=1.5, **γ**=1), though a **β** which is too high may also result in an overcompensation (top right; **β**=4, **γ**=1). Finally, hyperparameter **γ** is a smoothing kernel applied to the segmentation thresholds, to prevent the thresholding from varying abruptly from column-to-column. If sharper striping is observed, a smaller **γ** is used. Conversely, over-smoothing these segmentation thresholds (high **γ**) may also effectively negate adjustments to the thresholds made by **β** (last row).


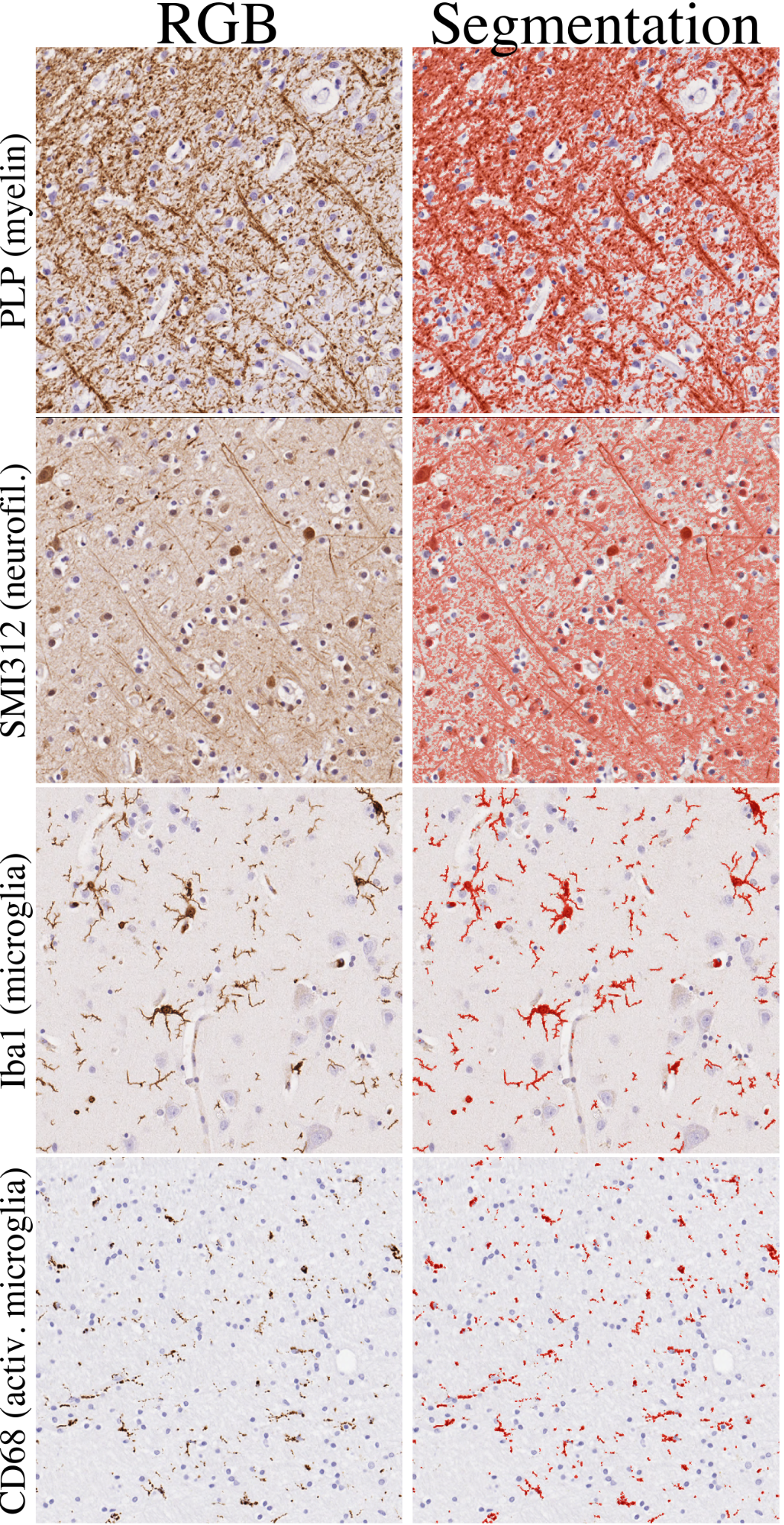


**Figure S3**: Example segmentations (right column; red overlay) produced from the automated SAF pipeline with optimised hyperparameters. The PLP (myelin) and SMI312 (neurofilament) patches were extracted from the WM and GM boundary in the visual cortex, the Iba1 (microglia) patch was sampled from hippocampal GM, and the CD68 (activated microglia) was taken from hippocampal WM.


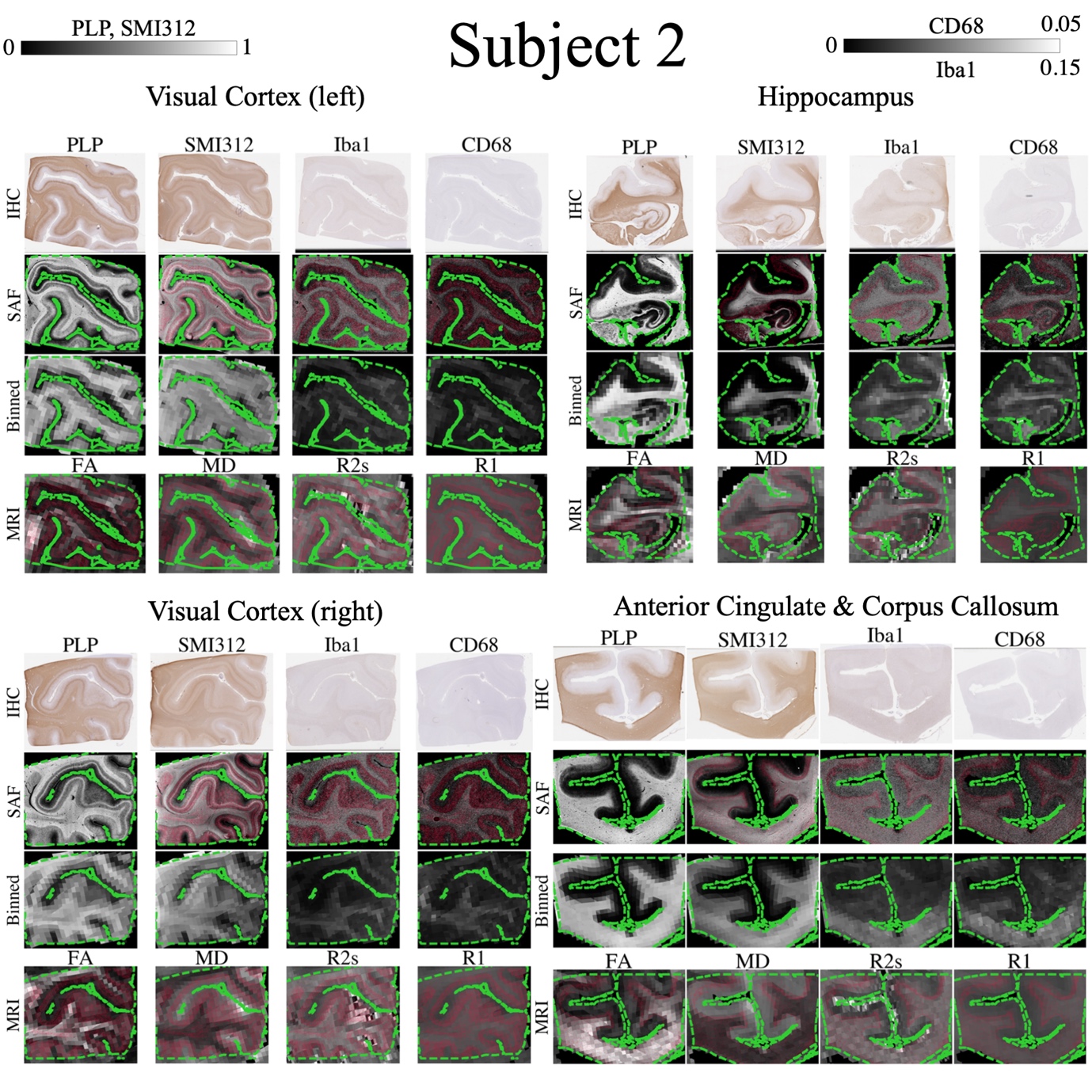


**Figure S4**: Registration evaluation for all brain regions in subject 2. Results are displayed as previously described in Figure 8. Again, we see close alignment of tissue borders and contours representing WM/GM contrast.


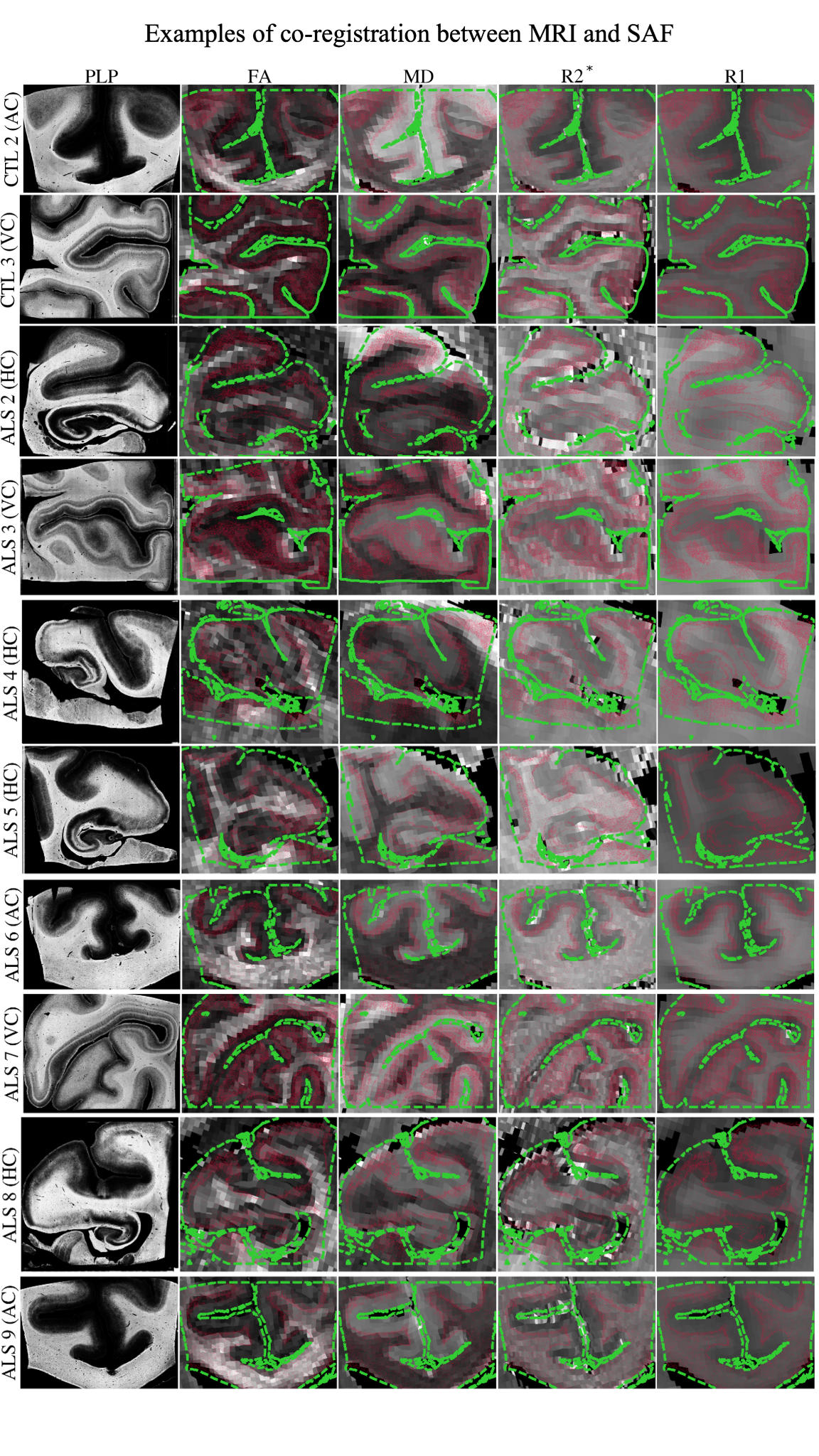


**Figure S5**: Registration evaluation for example brain regions extracted from 10 additional subjects (2 CTL, 10 ALS) (rows). In each brain region, contours of the tissue mask (green dashed) are overlaid on the co-registered MR parameter maps (second to fifth columns). The white and grey matter interface is shown in red. The tissue boundaries are closely aligned and the high registration accuracy enables us to perform meaningful voxelwise MRI-histology correlations.

**
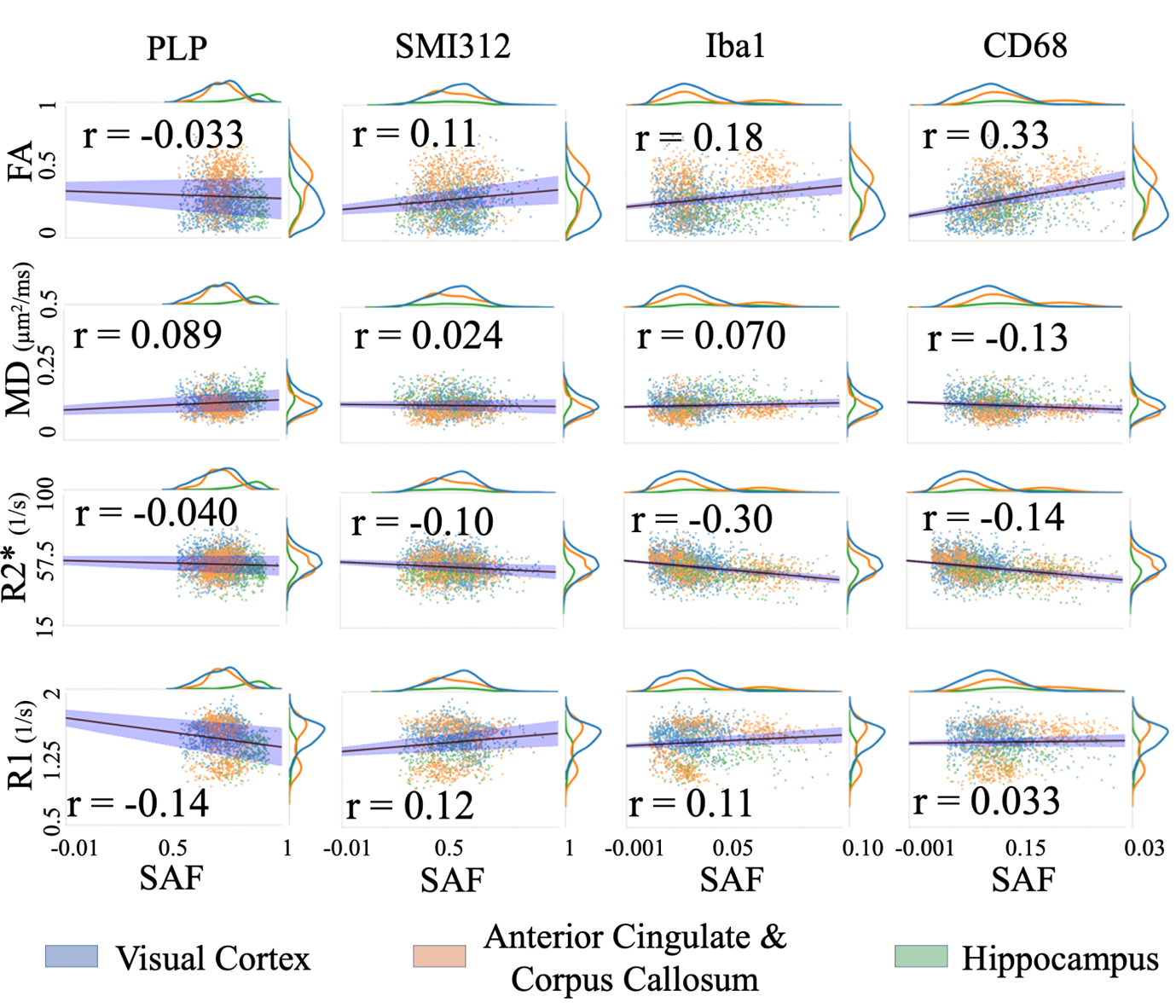
**

**Figure S6**: Correlating MR parameters (DTI FA, MD, R2* and R1) with IHC SAF in white matter only. The line of best fit (black line) and corresponding Pearson correlation coefficients, r, are overlaid. *PLP (myelin); SMI312 (neurofilaments); Iba1 (microglia); CD68 (activated microglia).*


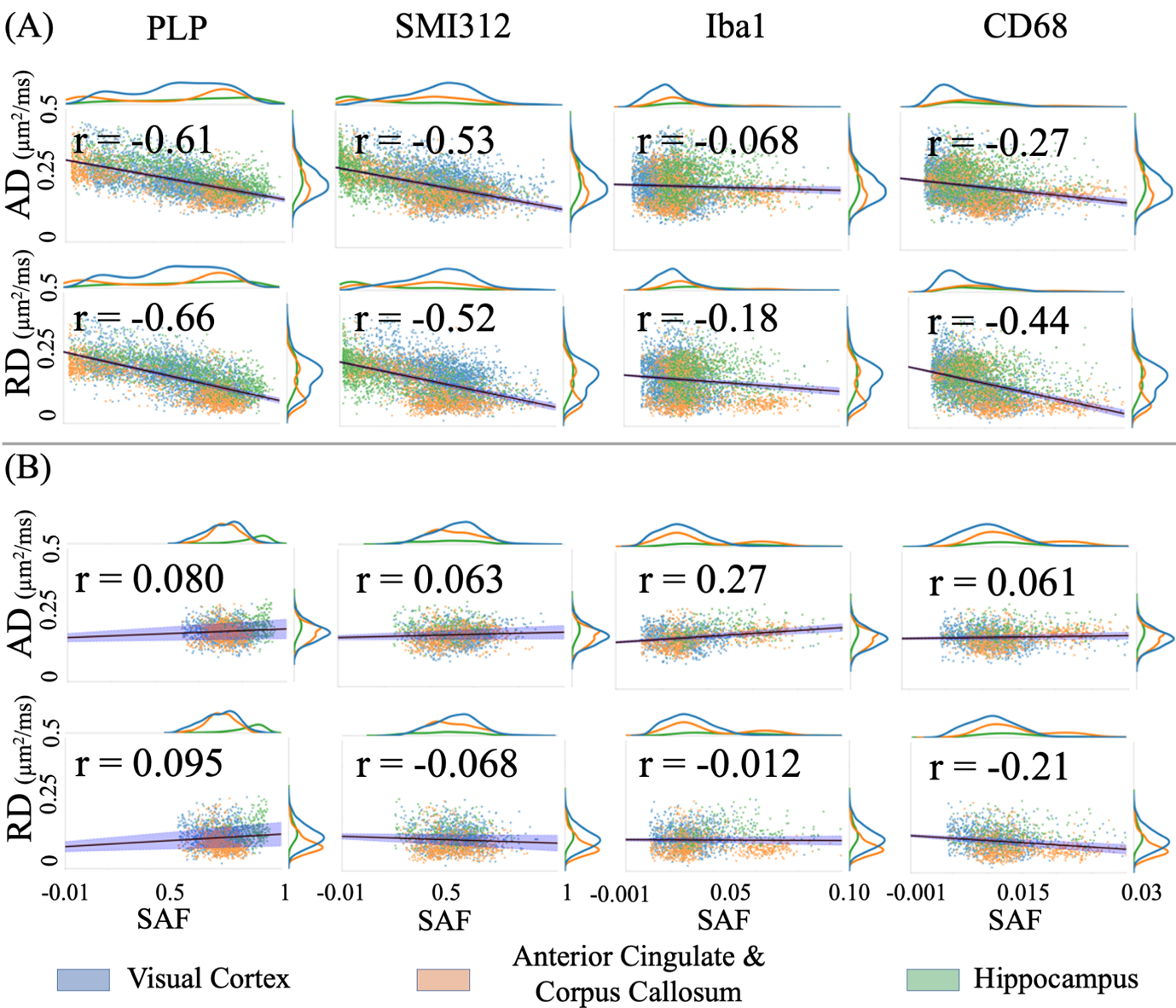


**Figure S7**: Correlating other DTI-derived parameters (AD, RD) with IHC SAF. This is done with all tissue voxels (A), and with voxels found the tissue’s WM only (B). Data is interpreted similarly to Figure S4.


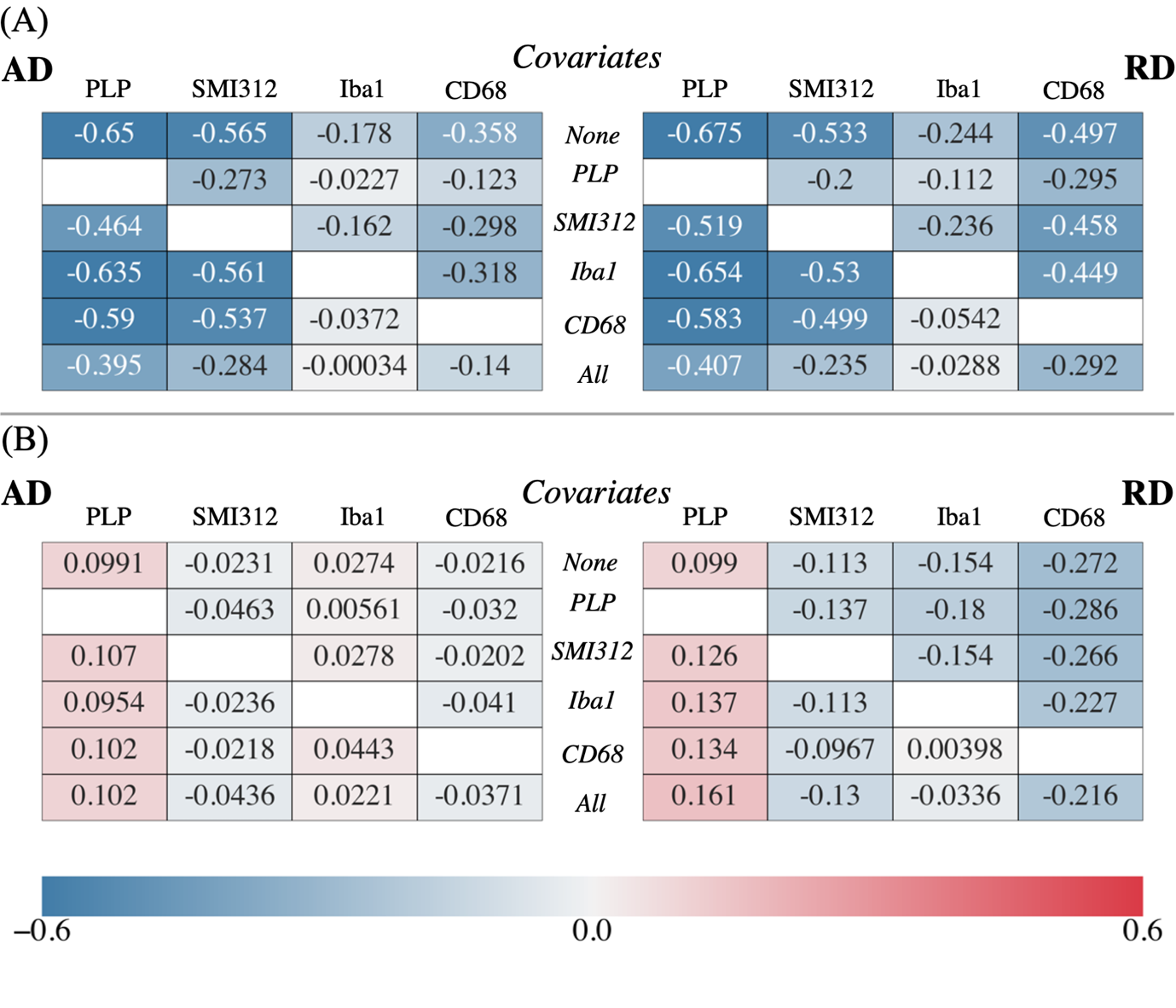


**Figure S8**: Partial correlation analysis between other DTI-derived parameters (AD, RD) and IHC stains for (A) both white and gray matter voxels and (B) for white matter voxels only. Each quadrant in either (A) or (B) is interpreted similarly to Figure 8. *PLP (myelin); SMI312 (neurofilaments); Iba1 (microglia); CD68 (activated microglia).*


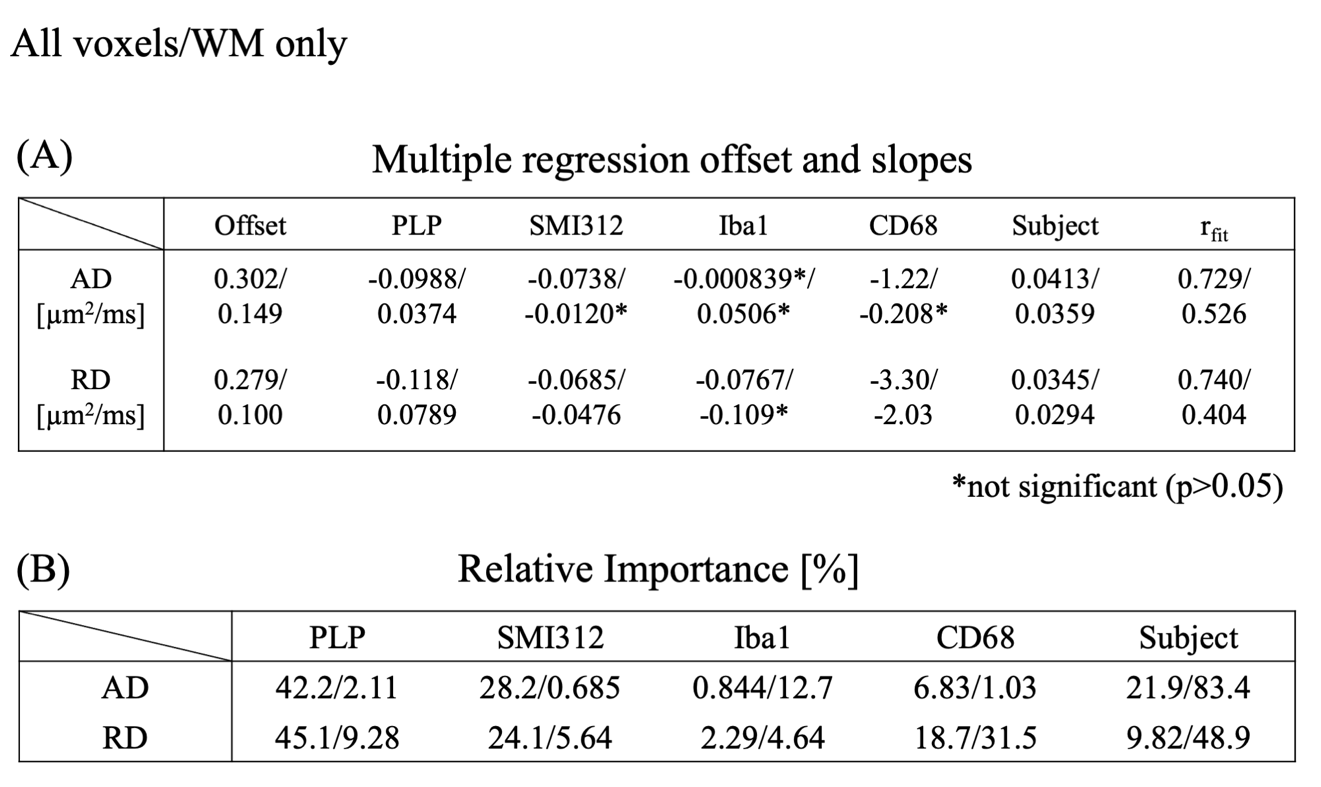


**Table S2**: Multiple linear regression predicting RD and MD using multiple IHC stains. Values are computed from all tissue voxels, or from voxels located in the tissue’s WM only. The table is interpreted similarly to Table 1.


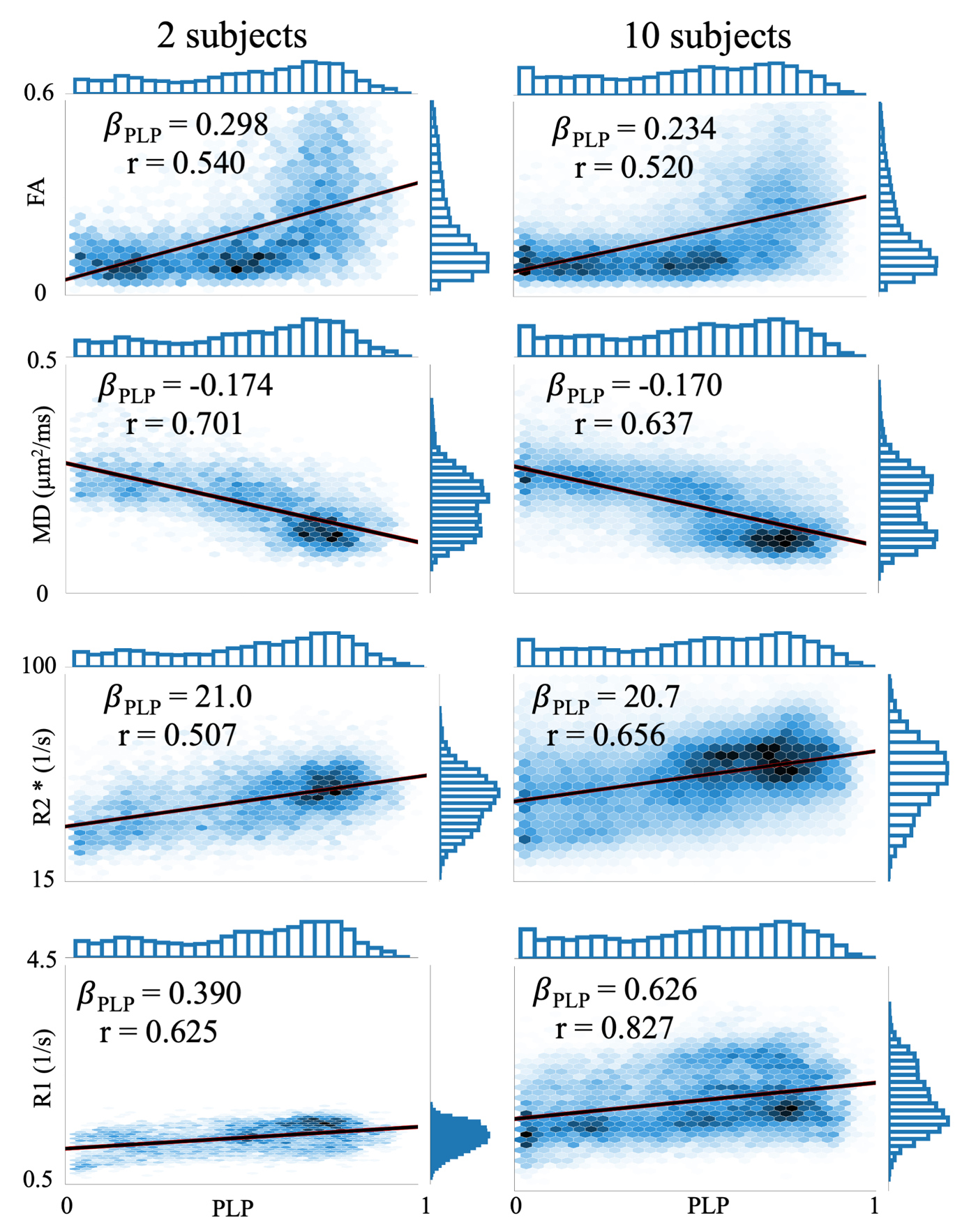


**Figure S9**: Density plots showing the relationship of different MR parameters (rows) with PLP (myelin) for both white and gray matter voxels. We compare the MRI-PLP regression coefficient (***β_PLP_***) derived from analysis across many subjects (CTL 2-3 & ALS 2-9; right column) with analysis across 2 subjects (CTL 1 & ALS 1; left column). The effect size, ***β_PLP_***, is highly consistent between the two datasets for FA, MD and R2*, but less so for R1.
